# Supplementary material for: Defects engineering simultaneously enhances activity and recyclability of MOFs in selective hydrogenation of biomass
Source: Nat Commun. 2022 Apr 19;13:2068. doi: 10.1038/s41467-022-29736-0 (PMC9018706; doi:10.1038/s41467-022-29736-0)
Supplement: Supplementary file 1 — Supplementary Information [file 41467_2022_29736_MOESM1_ESM.pdf]

Supplementary Information

Defects engineering simultaneously enhances activity and recyclability of MOFs in selective hydrogenation of biomass

Wenlong Xu[1,3], Yuwei Zhang[1,3], Junjun Wang[2,3], Yixiu Xu[1], Li Bian[1], Qiang, Ju[1], Yuemin Wang[2]* and Zhenlan Fang[1]*.

[1]Key Laboratory of Flexible Electronics (KLOFE) & Institute of Advanced Materials (IAM), Jiangsu National Synergetic Innovation Center for Advanced Materials (SICAM), Nanjing Tech University (NanjingTech), 30 South Puzhu Road, Nanjing 211816, P.R. China.

[2]Institute of Functional Interfaces, Karlsruhe Institute of Technology (KIT), 76344, Eggenstein-Leopoldshafen, Germany.

[3]These authors contributed equally: Wenlong Xu, Yuwei Zhang and Junjun Wang

*e-mail: yuemin.wang@kit.edu and iamzlfang@njtech.edu.cn

Table of contents

1. Experimental part: synthesis and characterization 3

1.1 Materials 3

1.2 Catalysis experiment 3

1.3 Characterization methods 3

1.4 Prepared samples 7

1.5 PXRD patterns 9

1.6 Thermal analysis 12

1.7 IR - spectroscopy data 14

1.8 NMR spectroscopy 16

1.9 Elemental analysis 20

1.10 XPS analysis of Ru@DEMOFs 21

1.11 Adsorption-desorption isotherms 23

1.12 STEM analysis 32

1.13 CO pulse chemisorption measurements 35

1.14 CO adsorption UHV-FTIR spectra 36

1.15 Temperature-dependent CO desorption UHV-FTIR spectra 37

1.16 XPS analysis of Au@DEMOFs 38

1.17 IR - spectroscopy data of DEMOFs and G-DEMOFs 39

1.18 SEM analysis of Ru@DEMOFs 40

2. Catalysis test of D-glucose selective hydrogenation 41

2.1 Optimize catalytic conditions of Ru@D_0, Ru@D_{1a}, Ru@D_{2a} toward D-glucose selective hydrogenation 41

2.1.1 Find out the optimal impregnated content of Ru in D_0, D_{1a} and D_{2a}. 41

2.1.2 Find out the optimal reaction temperature of Ru@D_0, Ru@D_{1a} and Ru@D_{2a} 43

2.1.3 Find out the optimal reaction pressure of Ru@D_0, Ru@D_{1a} and Ru@D_{2a} 45

2.1.4 Find out the optimal stirring rate of Ru@D_0, Ru@D_{1a} and Ru@D_{2a} 46

2.1.5 Find out the optimal reaction times of Ru@D_0, Ru@D_{1a-c} and Ru@D_{2a-c} 47

2.2 Blank Control Experiments 48

2.3 Kinetics Investigations 49

2.3.1 Determination of the reaction order of D-glucose selective hydrogenation to sorbitol 49

2.3.2. Calculation of Turnover Frequency (TOF) 52

2.4. The catalytic performances of Ru@D_0, Ru@$D_{1a'}$ and Ru@$D_{2a'}$ 54

3. Supplementary References 56

1. Experimental part: synthesis and characterization

1.1 Materials

All chemicals were purchased from commercial suppliers and used without further purification. All dried samples were stored under N_2 in a glovebox.

1.2 Catalysis experiment

The 50 g (25 wt%, 1.543 mol/L) D-glucose aqueous solution without any catalyst, in presence of Ru NPs, MOFs and Ru impregnated MOF catalysts (1 g), respectively, was transferred into a 100 mL stainless-steel high-pressure reactor. Before starting reaction, the reactor was purged with H_2 to 5.0 MPa, then degas to 1.0 MPa at room temperature, this process was repeated for three times to remove the air. The D-glucose aqueous solution, stirred with predetermined rates (600 or 800 rpm), was heated at the desired temperature (100, 120 or 140 ºC) under 5.0 MPa H_2 for a predetermined reaction time (ranging from 90 to 180 minutes), and then cooled to room temperature. Tiny amounts of aliquots were taken out every half an hour during reaction via a dip-tube inserted into the solution to test the activity of these catalysts. The separated reaction solution, obtained after removing the heterogeneous catalysts by centrifugation, was analyzed by HPLC to determine the conversion of D-glucose, selectivity and yield of sorbitol. To test the reusability of these catalysts, the catalysts were separated from the reaction solution, then washed with deionized water and ethanol, respectively, and finally dried in a vacuum oven at 80 ºC. The obtained catalysts after reaction were reused directly for the next run of biomass hydrogenation of D-glucose to form sorbitol.

1.3 Characterization methods

Powder X-ray diffraction (PXRD): PXRD patterns of all samples were recorded on a Rigaku Smartlab (3 KW) equipment with a Ni filter using Cu-Kα radiation (λ = 1.542 Å). The patterns were collected in reflectance of Bragg-Brentano geometry over a range of 2θ = 5 – 50º at room temperature.

Fourier transform infrared spectra (FTIR): FTIR patterns of all samples were recorded using a Bruker ALPHA FTIR under nitrogen atmosphere in the range 4000 – 400 cm^{-1}, placed in a N_2 glove-box.

Thermal gravimetric analysis (TGA): TGA curves of all samples with weight of ~ 10 mg were

recorded using a Mettler Toledo TGA instrument at atmospheric pressure under flowing of N_2 (99.9999%; flow rate = 50 mL/min), and the heating rate is ~10 ºC/min at a temperature ranging from 30 to 800 ºC.

N_2 adsorption/desorption measurements: the BET surface areas and pore size distributions, analyzed by both Barrett-Joyner-Halenda (BJH) and Density Functional Theory (DFT) method, were determined from the isothermal N_2 (99.9999%) adsorption/desorption curves of all samples with weight of ~100 mg, recorded by the Micromeritics 3 FLEX instrument at 78 K.

Scanning transmission electron microscopy (STEM): to analyze the morphology, size and dispersion of Ru NPs impregnated in the pristine and defect engineered MIL-100-Cr catalysts, STEM measurements of the powdered samples, deposited on porous amorphous carbon film supported by copper grids, were performed by a Talos F200X scanning transmission electron microscope at an acceleration voltage of 200 kV. STEM size distributions of Ru NPs can be approximated by Gaussin function.

Elemental analyzer (EA): EA were recorded on a Vario MACRO cube instrument to establish the C, H and N contents in Ru@$D_{1a\text{-}c}$.

Nuclear magnetic resonance spectroscopy (NMR): NMR were recorded on JNM-ECZ400S/L1 to determine the ratio of incorporated defective ligands (DLx, x =1, 2) to total ligands (TLs) in all DEMOFs and Ru@DEMOFs.

Inductively coupled plasma optical emission spectrometer (ICP-OES): ICP-OES were recorded on a Thermo Fischer ICAP7400 to set the content of Ru NPs in all Ru impregnated samples before and after catalysis, and to define the leaching content of chromium of all Ru impregnated samples after catalysis for three runs. All solid samples were digested by HNO_3/HCl (1:3) mixture at 180 ºC for 12 h before analysis.

Chemisorption (CS): CS measurements were performed on a Micromeritics 3 FLEX instrument using a dynamic pulse method to acquire the active surface areas (S_{act}). All samples were purged under hydrogen (99.9999%) flow at 150 ºC for 1 h, and then were cooled down to room temperature, subsequently, CO (99.9999%) pulses were injected until the calculated areas of consecutive pulses

were constant. The S_{act} of measured samples were calculated by assuming that one CO molecule was absorbed at the surface of one Ru atom and considering that Ru surface density is 1.64 x10^{19} atoms/m^2.[1]

UHV-FTIR spectroscopy and XPS: The ultra-high vacuum Fourier transformed infrared spectroscopy (UHV-FTIRS) and X-ray photoelectron spectroscopy (XPS) measurements were conducted with a sophisticated UHV apparatus combing a state-of-the-art FTIR spectrometer (Bruker Vertex 80v) and a multichamber UHV system (Prevac)[2-4]. This dedicated apparatus allows performing both IR transmission experiments on nanostructured powders and infrared reflection-absorption spectroscopy (IRRAS) measurements on well-defined model catalysts (single crystals and supported thin films). Both the optical path inside the IR spectrometer and the space between the UHV chamber as well as the spectrometer were evacuated to exclude the ambient molecule adoption, ensuring superior sensitivity and stability. The MOF sample (approximately 200 mg) was first pressed into an inert metal mesh which was mounted on an especially designed sample holder, and then activated in the UHV chamber at 500 K to remove all contaminants. Exposure to carbon monoxide (CO) was achieved using a leak-valve-based directional doser connected to a tube of 2 mm in diameter, which is terminated 3 cm from the sample surface and 50 cm from the hot-cathode ionization gauge. The IR experiments were carried out at temperatures as low as 110 K. All UHV-FTIR spectra were collected with 1024 scans at a resolution of 4 cm^{-1} in transmission mode, using a spectrum of the clean sample as a background reference.

The XPS experiments were carried out using a VG Scienta R4000 electron energy analyzer. The pass energy was fixed at 200 eV for all the measurements. A flood gun was applied to compensate for the charging effects. The binding energies were calibrated to the C1s line at 284.8 eV as a reference. The XP spectra were deconvoluted using the software Casa XPS with a Gaussian-Lorentzian mix function and Shirley background subtraction.

High performance liquid chromatography (HPLC): HPLC measurements were carried out on LC-20AT (Shimazu) instrument, equipped with a UV-Vis/refractive index detector (RID-10A) and an Aminex@HPX-87H ion exclusion column (300 mm × 7.8 mm) at 80 ºC, using pure water as the mobile phase (1.0 mL/min). The reaction solution, taken out by a tube inserted into the solution, was diluted with an equal volume of water. After being filtered over 0.22 μm filters, the diluted reaction solution was analyzed by HPLC. The conversion of D-glucose, selectivity and yield of sorbitol were obtained

from their corresponding concentrations after reaction, determined by the integral areas of their corresponding bands in the chromatograms and previously established calibration curves that were corrected by external standard method.

Scanning electron microscope (SEM): The SEM measurements were conducted by FEI (Quanta 200 FEG) SEM under a voltage of 5 kV to characterize the general morphologies of the Ru NPs impregnated MIL-100-Cr pristine MOF and defect engineered MOFs.

1.4 Prepared samples

Supplementary Table 1 List of synthesis details of samples.

DEMOF	DL*x*	Feeding mole ratio of DL*x* to TL (*z*)
D_0	/	/
$D_{1a'}$	DL1	5%
D_{1a}		10%
D_{1b}		30%
D_{1c}		50%
$D_{2a'}$	DL2	5%
D_{2a}		10%
D_{2b}		30%
D_{2c}		50%

Ru@DEMOF	DEMOFs	Feeding mass ratio of Ru element to MOF				
Ru@D_0	D_0	1%	1.5%	2%	2.5%	5%
Ru@$D_{1a'}$	$D_{1a'}$	2.5%				
Ru@D_{1a}	D_{1a}	1%	1.5%	2%	2.5%	5%
Ru@D_{1b}	D_{1b}	2.5%				
Ru@D_{1c}	D_{1c}					
Ru@$D_{2a'}$	$D_{2a'}$					
Ru@D_{2a}	D_{2a}	1%	1.5%	2%	2.5%	5%
Ru@D_{2b}	D_{2b}	2.5%				
Ru@D_{2c}	D_{2c}					

Au@DEMOFs	MOFs	Feeding mass ratio of Au element to MOF
Au@D_0	D_0	5%
Au@D_{1c}	D_{1c}	5%
Au@D_{2c}	D_{2c}	5%

D_0 represents pristine MIL-100-Cr, while $D_{1a'\text{-}c}$ and $D_{2a'\text{-}c}$ represent defect engineered MIL-100-Cr incorporated DL1 and DL2, respectively, with different feeding ratios (*z*) of defective linker (DL*x*, *x* = 1: 3,5-pyridinedicarboxylate; *x* = 2: *m*-phthalate) to total ligands (TL = DL*x* + parent linkers), ranging from 5% to 50%;

1.5 PXRD patterns

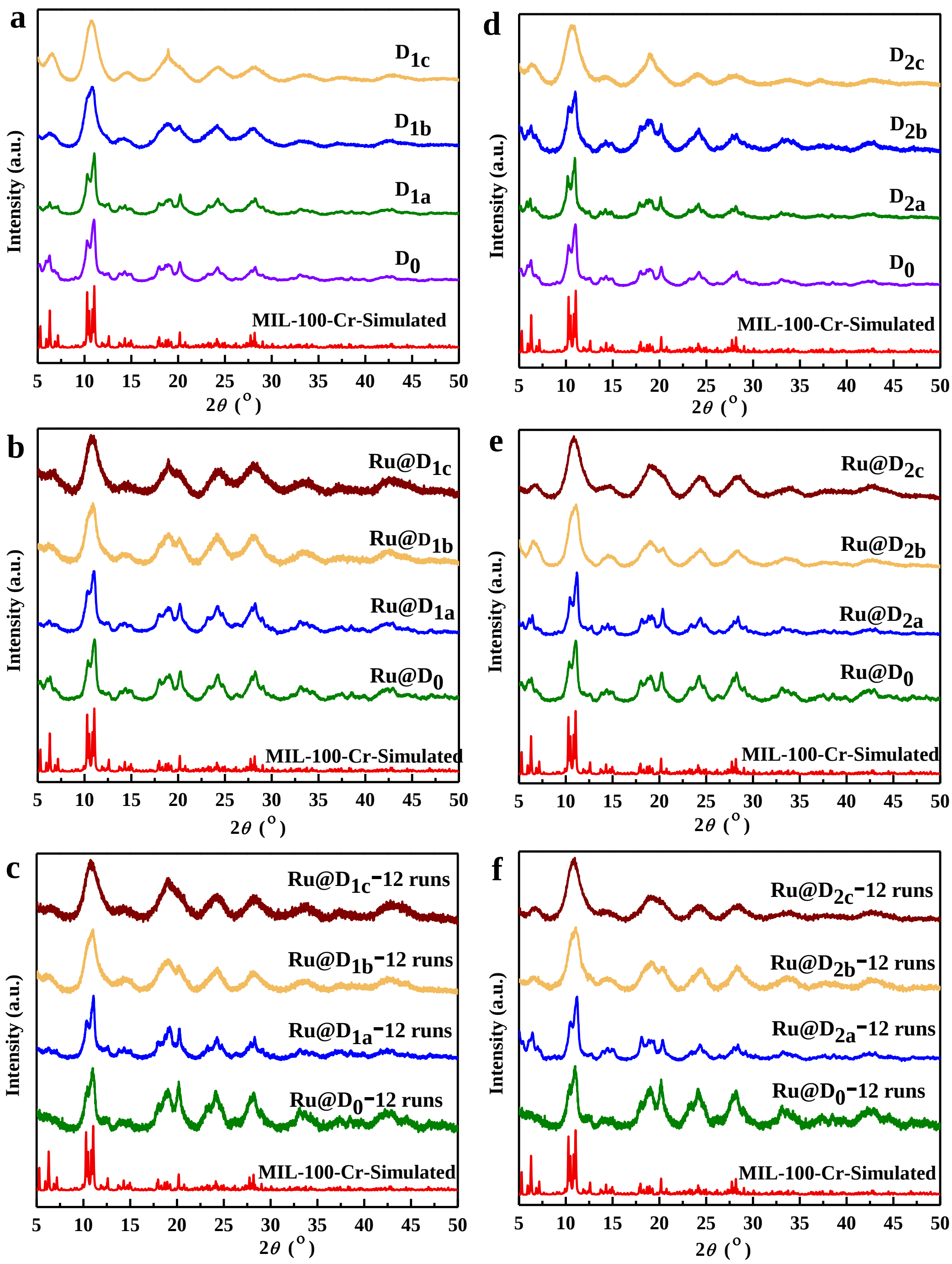

Supplementary Fig. 1 Powder X-ray diffraction patterns of D_0 and $D_{1a\text{-}c}$ (**a**), Ru@D_0 and Ru@$D_{1a\text{-}c}$ before catalysis (**b**) and after catalysis for 12 runs (**c**), D_0 and $D_{2a\text{-}c}$ (**d**), Ru@D_0 and Ru@$D_{2a\text{-}c}$ before catalysis (**e**) and after catalysis for 12 runs (**f**).

Supplementary Fig. 1a-f show that all the measured PXRD patterns of D_0, Ru@D_0, DEMOFs, and Ru@DEMOFs match well with the simulated XRD pattern derived from single crystal data of MIL-100 $Cr_3F(H_2O)_3O[C_6H_3\text{-}(CO_2)_3]_2{\cdot}nH_2O$ (n ~ 28)[5], demonstrating that these samples maintain the

framework of MIL-100-Cr, and the amount of any impurities in these samples is trace. The distinguishable fine peaks in PXRD patterns of dried powder samples D_0, D_{1a}, $D_{2a\text{-}b}$, Ru@D_0, Ru@D_{1a} and Ru@D_{2a} before and after catalysis for 12 runs indicate that they have relatively large particle sizes with good crystalline. These results confirm that the cationic framework of MIL-100-Cr has good tolerance to the incorporated DL*x* (*x* = 1: 3,5-pyridinedicarboxylate, *x* = 2: m-phthalate). For both types of DEMOF and Ru@DEMOFs, the fine peaks gradually disappeared and merged into broad bands along with increasing the feeding ratio (*z*) of DL*x* to TL ($x = 1$, $z \geq 30\%$; $x = 2$, $z \geq 50\%$), attributed to the decrease of particle size (Supplementary Fig. 33)[6]. After Ru impregnation, the presence of broad bands accompanying the disappearance of fine peaks in the PXRD pattern of D_{2b} illustrates that the process of Ru impregnation results in decreases of particle sizes of D_{2b}[7].

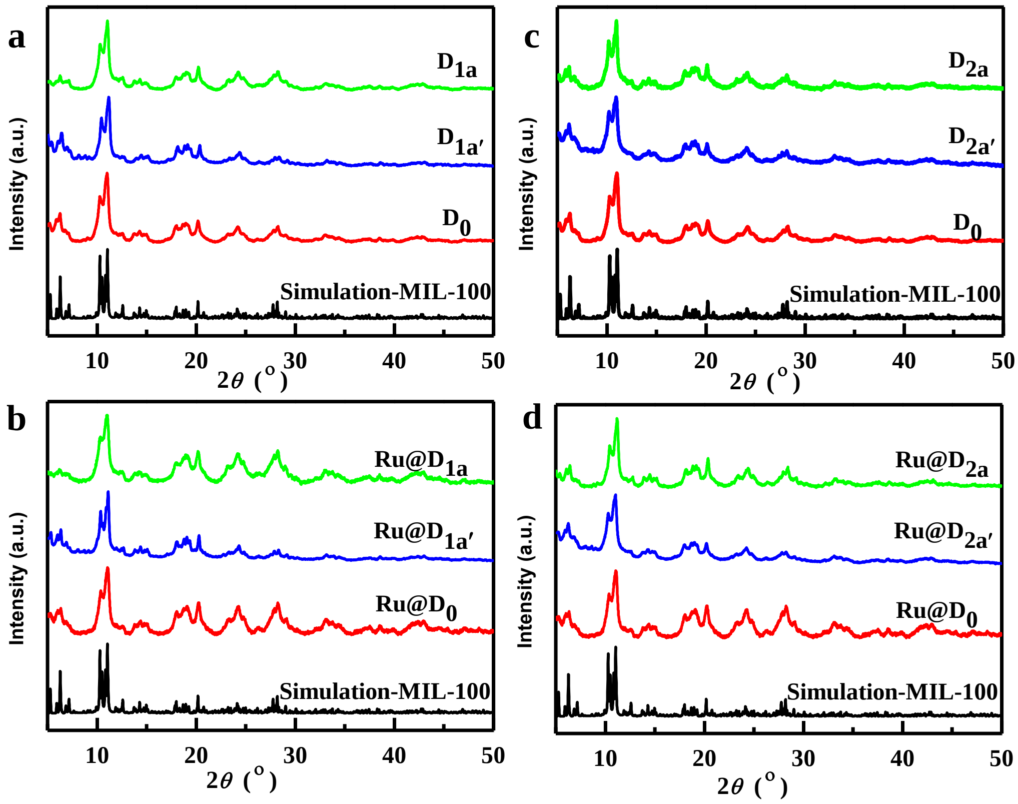

Supplementary Fig. 2 Powder X-ray diffraction patterns of D_0, $D_{1a'}$ and D_{1a} (**a**), Ru@D_0, Ru@D_{1a}′ and Ru@D_{1a} (**b**), D_0, $D_{2a'}$ and D_{2a} (**c**), Ru@D_0, Ru@$D_{2a'}$ and Ru@D_{2a} (**d**).

Supplementary Fig. 2a-d show that all the measured PXRD of dried powder samples $D_{1a'}$, $D_{2a'}$, Ru@$D_{1a'}$ and Ru@$D_{2a'}$ match well with the simulated XRD pattern derived from a single crystal of MIL-100-Cr, confirming the maintenance of frameworks for these samples. The distinguishable fine peaks in PXRD patterns of these dried powder samples indicate that they have relatively large particle sizes with good crystallinity.

1.6 Thermal analysis

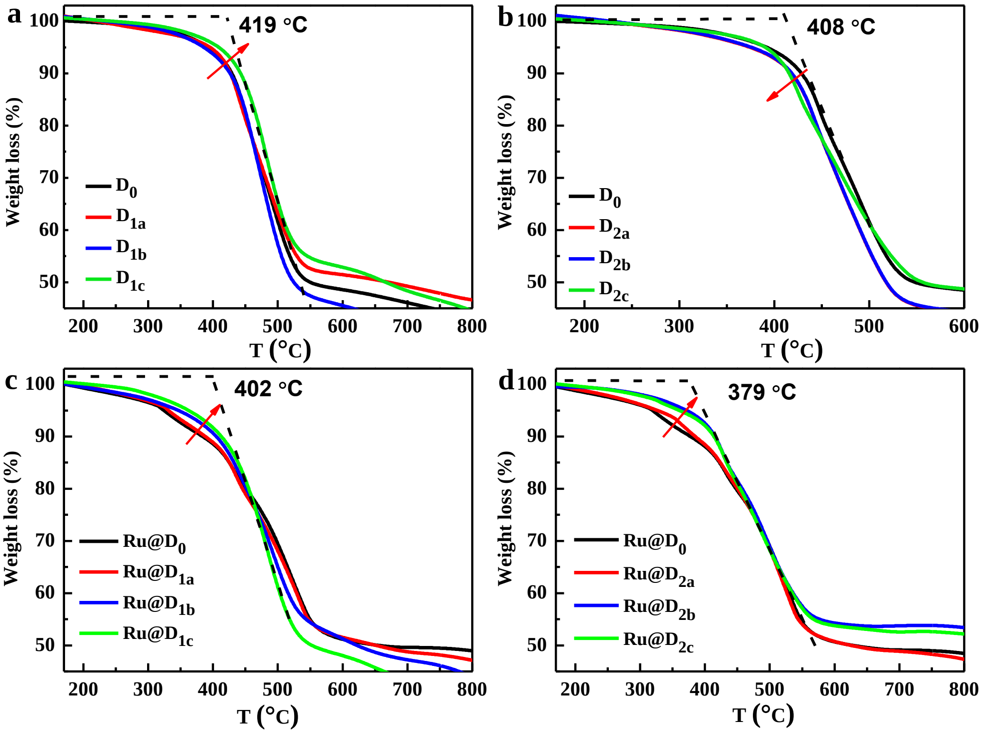

Supplementary Fig. 3 TGA curves of D_0 and $D_{1a\text{-}c}$ dry samples (**a**), D_0 and $D_{2a\text{-}c}$ dry samples (**b**), Ru@D_0 and Ru@$D_{1a\text{-}c}$ dry samples (**c**), as well as Ru@D_0 and Ru@$D_{2a\text{-}c}$ dry samples (**d**).

All TGA curves of these samples are quite similar to that of D_0, illuminating that they maintain the framework of MIL-100-Cr. The highest thermal stability of these four types of samples $D_{1a\text{-}c}$, $D_{2a\text{-}c}$, Ru@$D_{1a\text{-}c}$ and Ru@$D_{2a\text{-}c}$ are up to 419, 408, 402 and 379 ºC, respectively, as shown in Supplementary Fig. 3a-d. The comparison of these TGA curves shows that the thermal stability of $D_{1a\text{-}c}$ is enhanced with increasing feeding ratio (z) of DL*x* to TL, being contrast to that of $D_{2a\text{-}c}$. Moreover, the thermal stability of the Ru impregnated $D_{1a\text{-}c}$ catalysts is higher than that of corresponding Ru impregnated $D_{2a\text{-}c}$, primarily attributed to the weak coordination bond between the Cr-CUSs and N atoms of DL1. These results demonstrate the successful incorporation of DL*x* and different modification functions of type-A and type-B defects on the framework of MIL-100-Cr. The evolutions of thermal stability for these two kinds of Ru NPs impregnated catalysts Ru@$D_{1a\text{-}c}$ and Ru@$D_{2a\text{-}c}$ are the same as that of $D_{1a\text{-}c}$. Noticeably, all these Ru impregnated MOFs catalysts show overall lower thermal stability than the respective corresponding MOFs supporters, illuminating that the Ru impregnation process has critical effects on the framework of MIL-100-Cr.

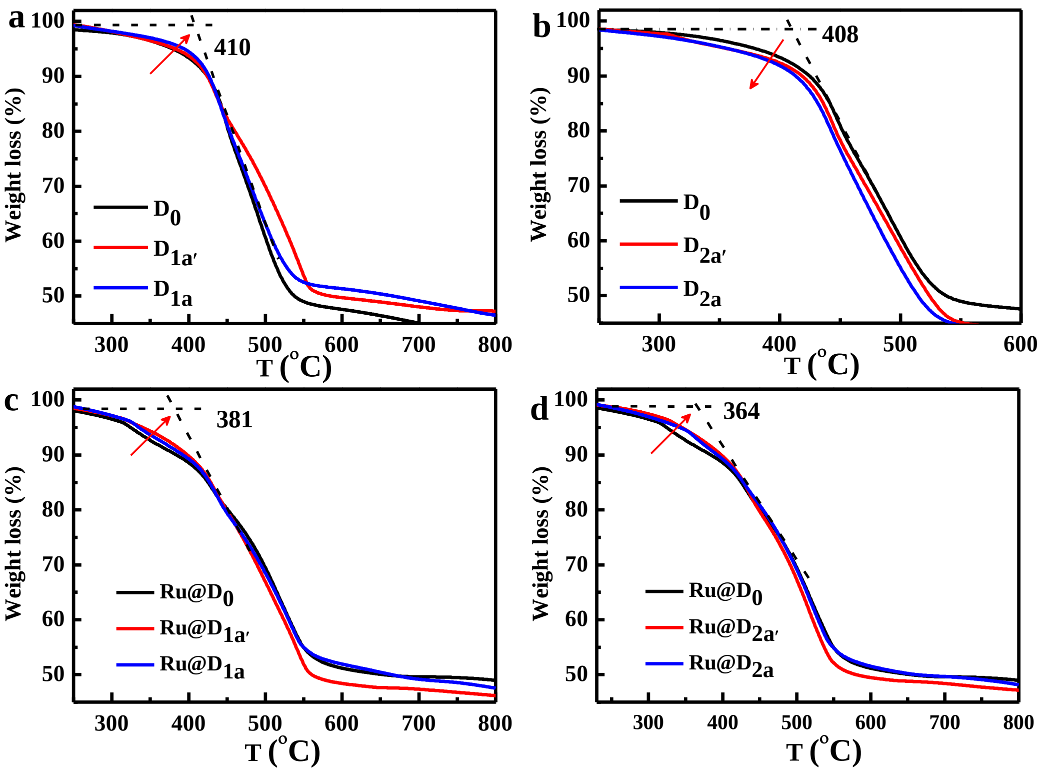

Supplementary Fig. 4 TGA curves of D_0, $D_{1a'}$ and D_{1a} dry samples (**a**), D_0, $D_{2a'}$ and D_{2a} dry samples (**b**), Ru@D_0, Ru@$D_{1a'}$ and Ru@D_{1a} dry samples (**c**), as well as Ru@D_0, Ru@$D_{2a'}$ and Ru@D_{2a} dry samples (**d**).

All TGA curves of $D_{1a'}$, $D_{2a'}$, Ru@$D_{1a'}$ and Ru@$D_{2a'}$ are quite similar to that of D_0, illuminating that they maintain the framework of MIL-100-Cr. As shown in Supplementary Fig. 4a-d, the evolution trends of thermal stability of DEMOFs containing type-A defects, DEMOFs containing type-B defects, Ru NPs impregnated DEMOFs containing type-A defects and Ru NPs impregnated DEMOFs containing type-B defects with feeding ratio (z) ranging from 0% to 10% are consistent with that of feeding ratio (z) ranging from 0% to 50%, respectively (Supplementary Fig. 3).

1.7 IR - spectroscopy data

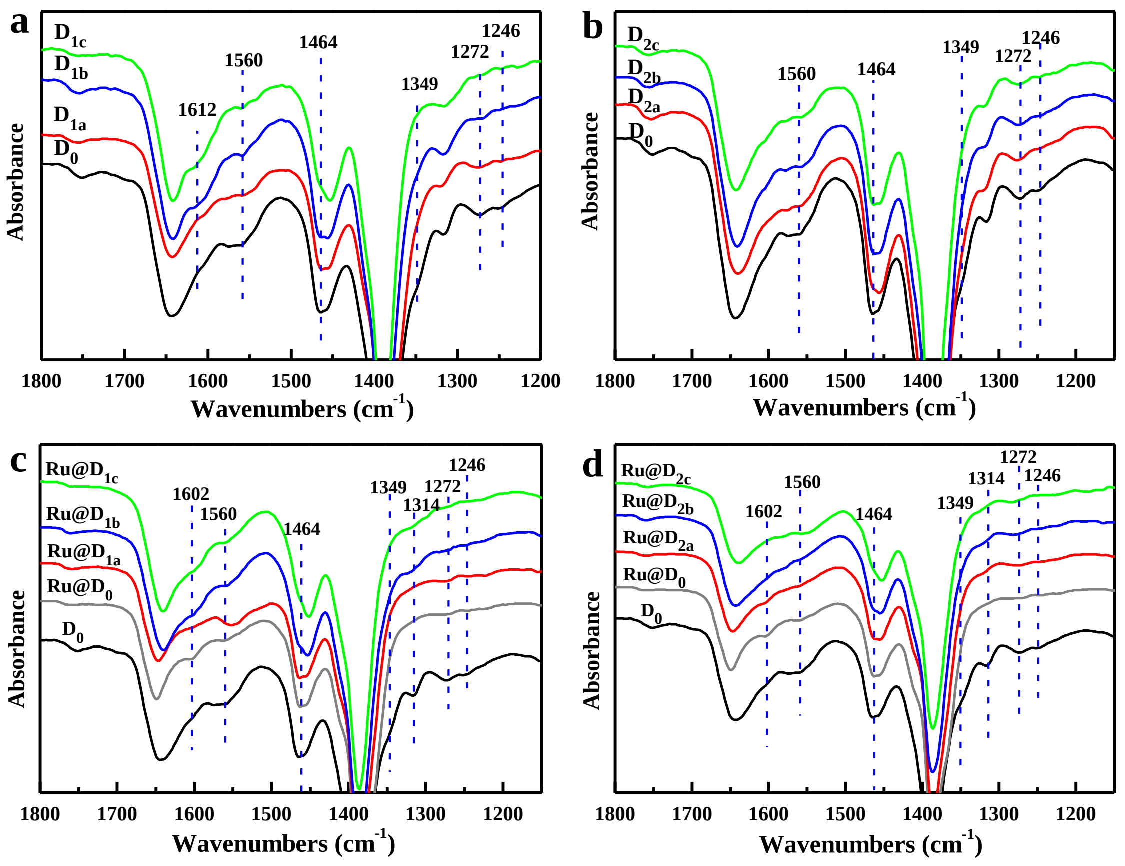

Supplementary Fig. 5 FT-IR spectra of the dry, activated DEMOFs $D_{1a\text{-}c}$ (**a**) and $D_{2a\text{-}c}$ (**b**) in comparison with D_0, and activated Ru@$D_{1a\text{-}c}$ (**c**), Ru@$D_{2a\text{-}c}$ (**d**) in comparison with Ru@D_0.

As shown in Supplementary Fig. 5a-d, the IR spectra of all DEMOFs, Ru@D_0 and Ru@DEMOFs feature the typical band shape of the parent D_0, indicating that all DEMOFs and the Ru impregnated MOFs catalysts maintain the framework of MIL-100-Cr, and no significant unreacted ligands remain in their pores after being washed and activation. Upon increasing the feeding ratio of DL*x* to TL, the shoulder bands at 1612 cm^{-1}, assigned to the asymmetric stretching vibration of C = O on DL1[8], in the FTIR spectra of $D_{1a\text{-}c}$ become gradually distinct, the band at 1464 cm^{-1} (the stretching vibration of C = C on aromatic ring[9]) stepwise shifts to lower frequency, meanwhile the bands at 1349 (symmetrical stretching vibration peak of C=O of carboxyl group on L0), 1272 and 1246 cm^{-1} (symmetrical stretching vibration peak of C-O on L0)[10] become indistinct for both two kinds of DEMOFs as well as their Ru impregnated catalysts. These results confirm the successful incorporation of DL*x* in the framework of these samples. The shoulder band at 1602 cm^{-1}, assigned to the asymmetric stretching vibration of C = O of L0[11], becomes more discriminable in the IR spectrum of Ru@D_0 by comparison

with that in D_0, while the bands at 1314 cm^{-1} disappear in all Ru impregnated MOFs catalysts but are visible in all MOFs supporters (D_{1a-c} and D_{2a-c}). These results demonstrate that the Ru impregnation process plays a crucial role in the modification of the framework of MIL-100-Cr.

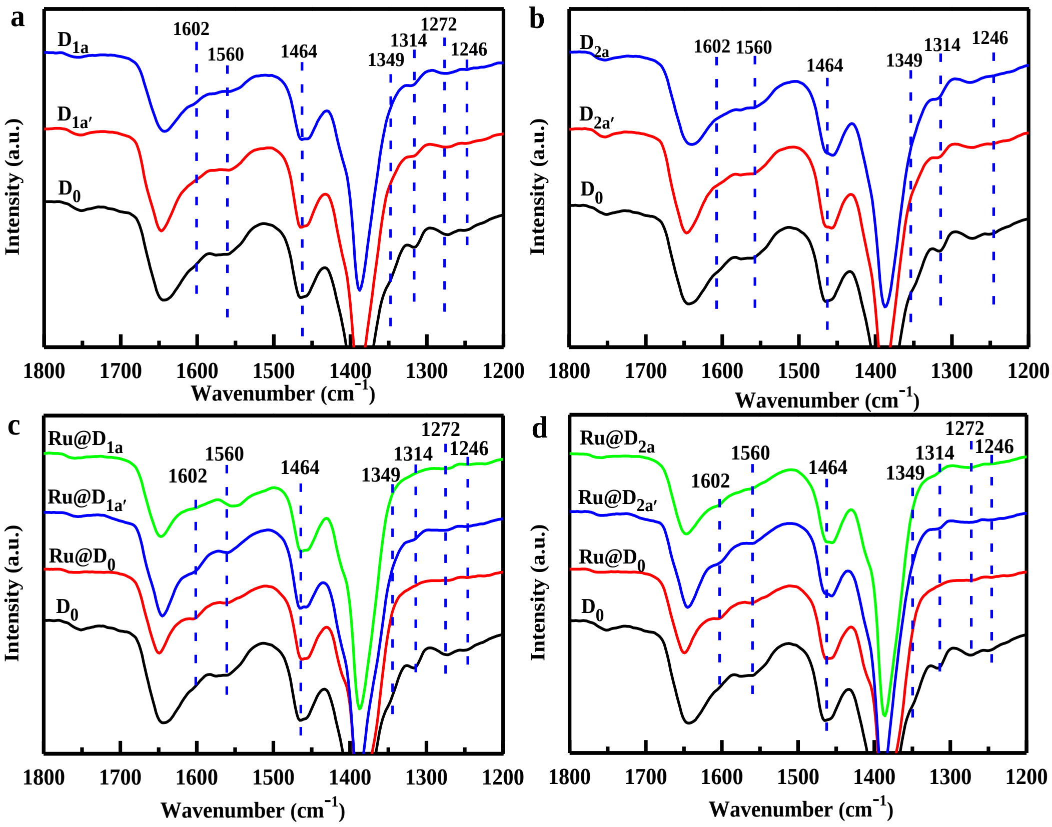

Supplementary Fig. 6 FT-IR spectra of the dry, activated DEMOFs, $D_{1a'}$, D_{1a} (**a**) and, $D_{2a'}$, D_{2a} (**b**) in comparison with D_0, and activated Ru@$D_{1a'}$, Ru@D_{1a} (**c**), Ru@$D_{2a'}$, Ru@D_{2a} (**d**) in comparison with D_0 and Ru@D_0.

As shown in Supplementary Fig. 6a-d, the IR spectra of $D_{1a'}$, $D_{2a'}$, Ru@$D_{1a'}$ and Ru@$D_{2a'}$ feature the typical band shape of parent D_0, indicating that they maintain the framework of MIL-100-Cr, and no significant unreacted ligands remain in their pores after being washed and activation. The evolution trends of FTIR spectra of the DEMOFs containing type-A defects, DEMOFs containing type-B defects, Ru NPs impregnated DEMOFs containing type-A defects and Ru NPs impregnated DEMOFs containing type-B defects with feeding ratio (z) ranging from 0% to 10% are consistent with that of feeding ratio (z) ranging from 0% to 50%, respectively (Supplementary Fig. 5).

1.8 NMR spectroscopy

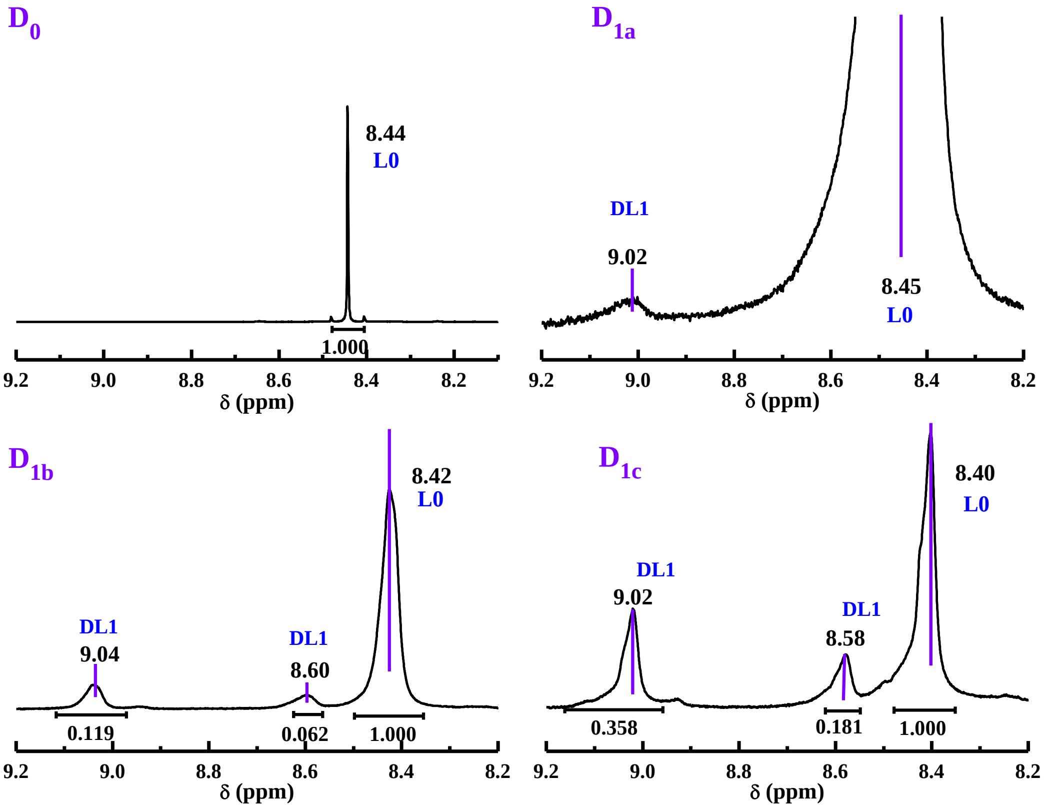

Supplementary Fig.7 ^{1}H NMR spectra of the digested dry samples of D_0 and $D_{1a\text{-}c}$.

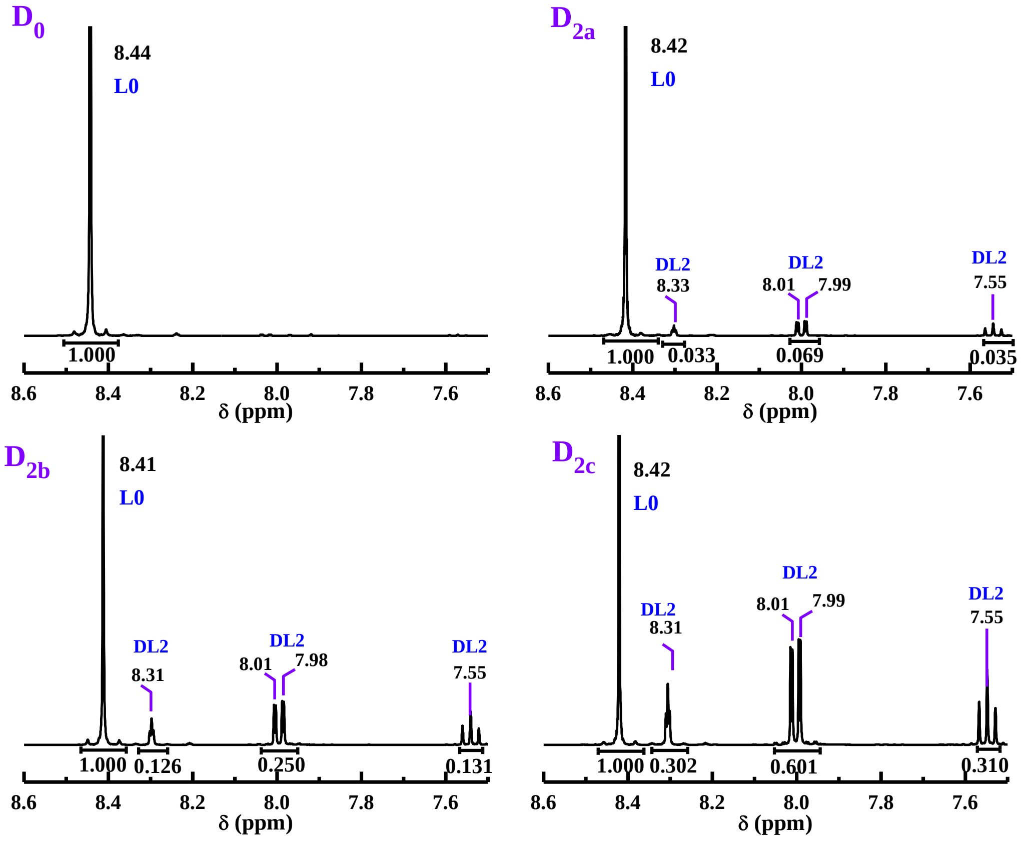

Supplementary Fig. 8 ^{1}H NMR spectra of the digested dry samples of D_0 and $D_{2a\text{-}c}$.

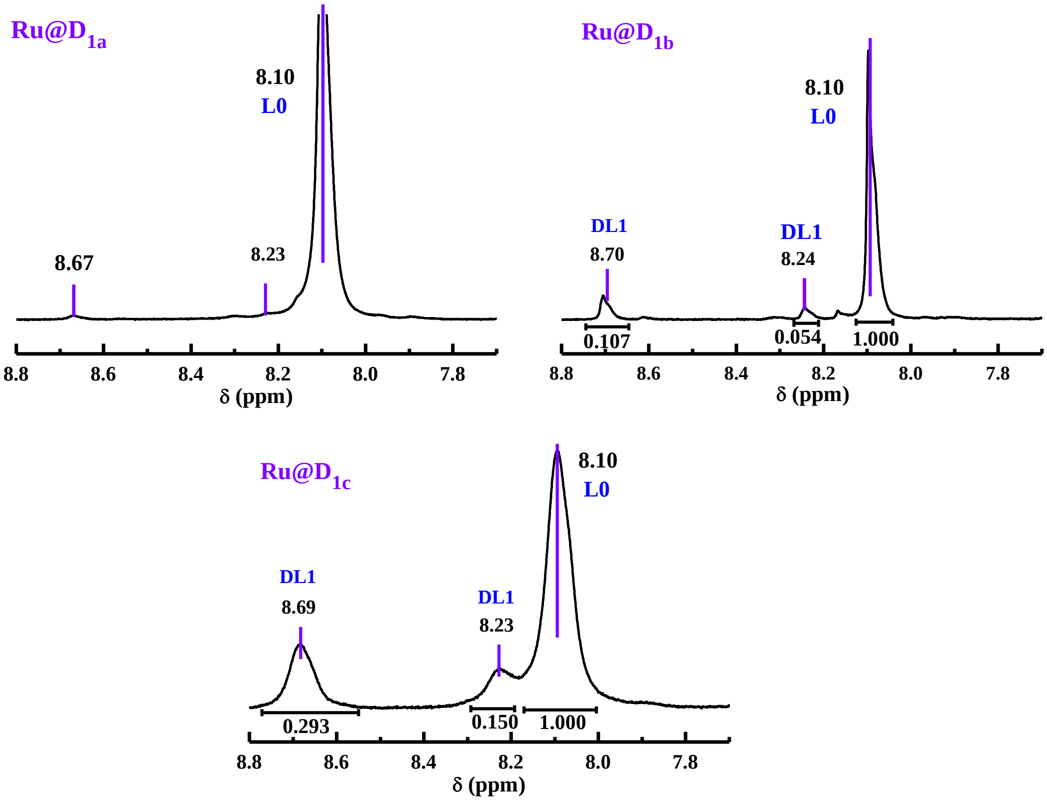

Supplementary Fig. 9 ^{1}H NMR spectra of the digested dry samples of Ru@$D_{1a\text{-}c}$.

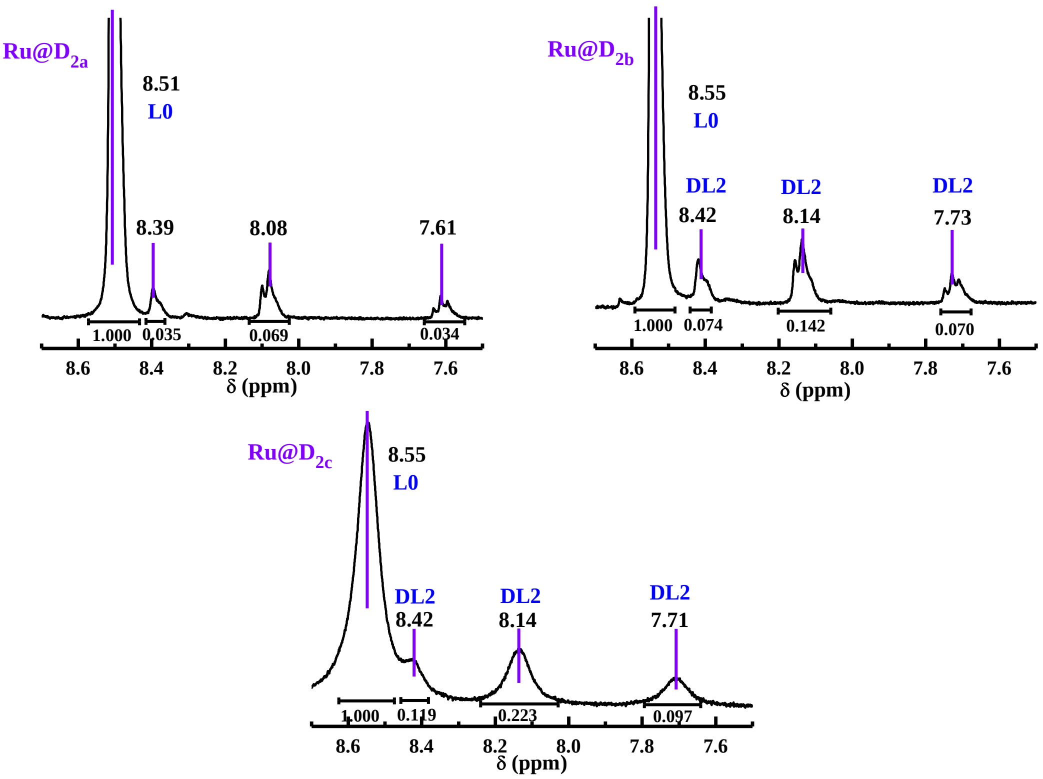

Supplementary Fig. 10 ^{1}H NMR spectra of the digested dry samples of Ru@D_{2a-c}.

Note: Before NMR measurement, all samples were firstly digested by 1 M NaOH solution. To reduce the shielding effect of the paramagnetic Cr ions on NMR, the $Cr(OH)_3$ precipitation was removed from the obtained digested solution. The presence of DL1 in D_{1a} and Ru@D_{1a} can be confirmed by ^{1}H NMR, but the ratio of incorporated DL1 to TL in them cannot be precisely determined by ^{1}H NMR due to the low signal to noise ratio of the resonances assigned to the aromatic C-H groups of DL1 induced by its low concentration.

Supplementary Table 2 The mole ratio of the incorporated defective linker (DL*x*) to that of total linkers (TL) (i.e., L*x* + btc), obtained from ^{1}H NMR spectra.

Sample	Ratio of L*x* to TL (%)	Sample	Ratio of L*x* to TL (%)	Ratio of leaching DL*x* (%)
D_{1a}	/	Ru@D_{1a}	/	/
D_{1b}	15.3	Ru@D_{1b}	13.9	1.4
D_{1c}	35.0	Ru@D_{1c}	30.7	4.3
D_{2a}	9.3	Ru@D_{2a}	9.3	0
D_{2b}	27.5	Ru@D_{2b}	17.7	9.8
D_{2c}	47.6	Ru@D_{2c}	24.8	22.8

The peak attributed to the aromatic C-H groups of parent ligand L0 (btc^{3-}) in the ^{1}H NMR spectra of the digested dry, activated D_0, $D_{1a\text{-}c}$, $D_{2a\text{-}c}$ samples are at ~8.42 ppm, while that of the digested dry, activated Ru@$D_{1a\text{-}c}$ and Ru@$D_{2a\text{-}c}$ samples are at ~8.10 and ~8.55 ppm, respectively. The peak shifts can be attributed to the presence of Ru NPs and Cr anions. For each kind of DEMOFs and Ru@DEMOFs, the ratio of integration of the resonances, assigned to the aromatic C-H groups of DL*x* to that of the peak attributed to the aromatic C-H groups of btc^{3-} increases upon increasing feeding mole ratio of DL*x* to TL (z), indicating that increasing z leads to the increase of incorporating concentration of DL*x* in DEMOFs. These results demonstrate that the incorporated concentration of DL*x* can be tuned by changing the feeding mole ratios of DL*x* to TL. The contents of DL*x* in DEMOFs are more than corresponding Ru@DEMOFs when $z \geq 30\%$, as shown in Supplementary Table 2, illuminating that the Ru impregnation process leads to leaching of DL*x* from the framework of DEMOFs, and the leaching degree of DL*x* increase upon increasing the incorporated DL*x*. Noticeably, the incorporated DL2 of low concentration can maintain consistency in the framework of D_{2a} during Ru impregnation process.

1.9 Elemental analysis

Supplementary Table 3 The defined formulas for the dry, activated D_{1a}, Ru@D_0 and Ru@D_{1a-c} samples by EA

Name	N (wt%)	C (wt%)	H (wt%)	Ratio of DL1 to TL (%)	Formula	Ratio of leaching DL*x*
D_{1a}	0.335	34.31	4.111	7.41	$[Cr_3F_y(H_2O)_3O(btc)_{1.852}(DL1)_{0.148}]\cdot nH_2O$	/
Ru@D_{1a}	0.337	34.35	4.115	7.45	$Ru_m[Cr_3F_y(H_2O)_3O(btc)_{1.851}(DL1)_{0.149}]\cdot nH_2O$	0.04%
Ru@D_{1b}	0.959	37.98	3.959	18.65	$Ru_m[Cr_3F_y(H_2O)_3O(btc)_{1.627}(DL1)_{0.373}]\cdot nH_2O$	/
Ru@D_{1c}	1.499	34.65	4.194	31.05	$Ru_m[Cr_3F_y(H_2O)_3O(btc)_{1.379}(DL1)_{0.621}]\cdot nH_2O$	/

To verify the ratio of incorporated DL1 to TL (%) in Ru@D_{1b-c} obtained from ^{1}H NMR spectra and to get the ratios of incorporated DL1 to TL in D_{1a} and Ru@D_{1a}, which cannot be precisely determined by ^{1}H NMR due to their low concentration, the EA has been conducted on D_{1a} and Ru@D_{1a-c} as the ratio of DL1 to TL in these samples can also be calculated based on the N content. Overall, the ratios of DL1 to TL for obtained from EA are consistent with that of D_{1b-c} and Ru@D_{1b-c} obtained from ^{1}H NMR spectra, demonstrating the reliability of the ratios of DL*x* to TL and the accurateness of the composition of all samples determined from ^{1}H NMR. Similar to DL2, the low leaching amount of DL1 for Ru@D_{1a} compared with D_{1a} illuminates that the incorporated DL1 of low concentration can maintain consistency in the framework of D_{1a} during Ru impregnation process.

1.10 XPS analysis of Ru@DEMOFs

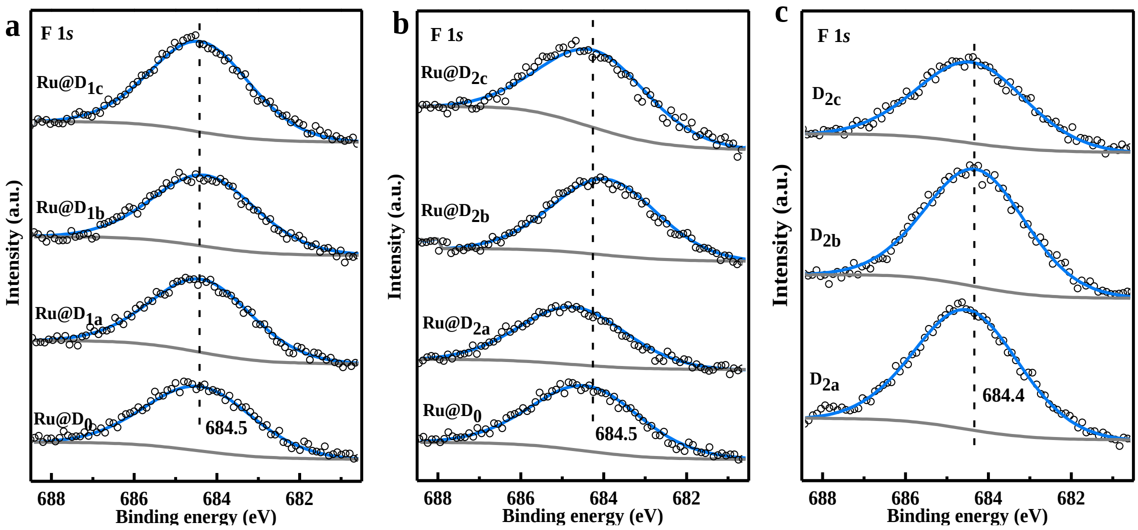

Supplementary Fig. 11 XPS spectra of F 1*s* region for Ru@D_0 and Ru@$D_{1a\text{-}c}$ (**a**), Ru@D_0 as well as Ru@$D_{2a\text{-}c}$ (**b**), and $D_{2a\text{-}c}$ (**c**).

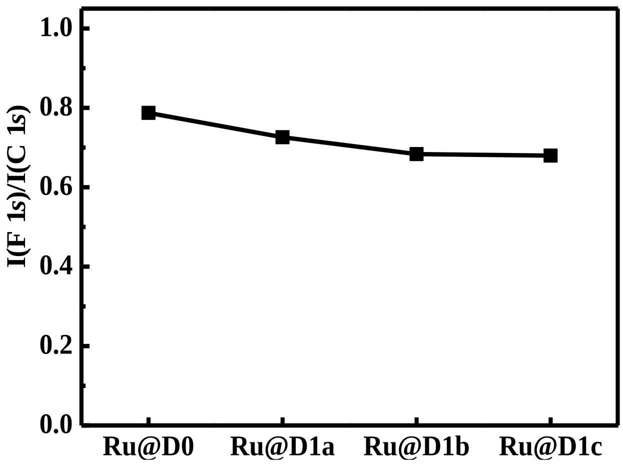

Supplementary Fig. 12 The ratios of the integrated peak areas of the F 1*s* band to that of C 1*s* band in the high-resolution XPS spectra of Ru@D_0 and Ru@$D_{1a\text{-}c}$.

The bands of F 1*s* are centered at the binding energy of ~684.4 eV (Supplementary Fig. 11), and the ratios of the integral area of the F 1*s* band to that of C 1*s* band for the selected catalysts (Ru@$D_{1a\text{-}c}$) are all lower than that of Ru@D_0 (Supplementary Fig. 12), excluding the possibility that the missing charge resulting from the incorporated DL*x* are compensated by increasing the concentration of F^- counterions.

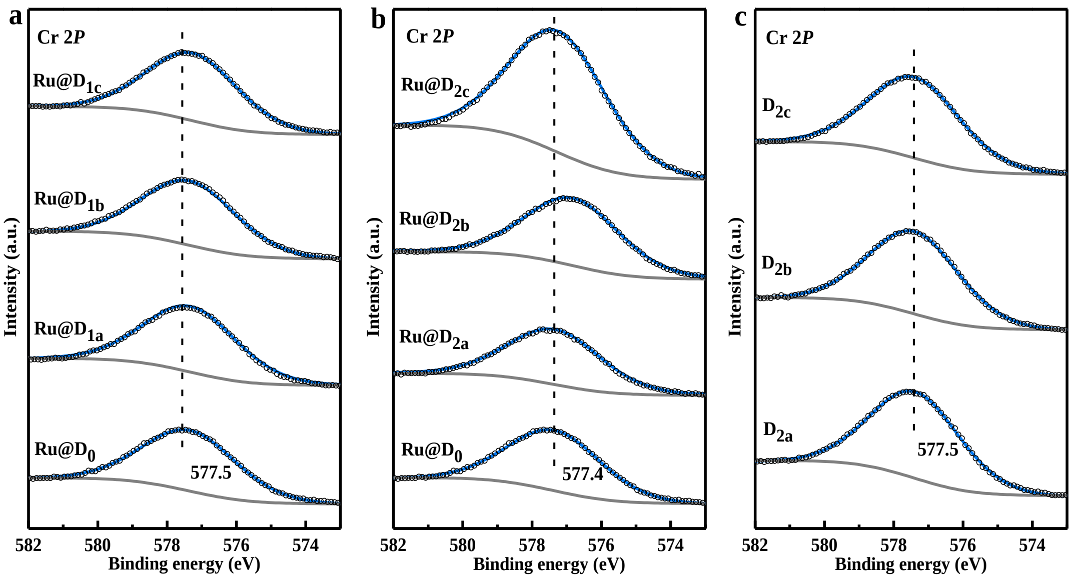

Supplementary Fig. 13 High-resolution XPS spectra of Cr 2*p* region for Ru@D_0 and Ru@$D_{1a\text{-}c}$ (**a**), Ru@D_0 and Ru@$D_{2a\text{-}c}$ (**b**), $D_{2a\text{-}c}$ (**c**).

The bands for Cr^{3+} and $Cr^{\delta+}$ ($\delta < 3$) cannot be distinguished in the high-resolution XPS spectra of Cr 2*p*, but the presence of $Cr^{\delta+}$ ($\delta \leqslant 3$) nodes could be clearly confirmed by UHV-FTIR spectra.

1.11 Adsorption-desorption isotherms

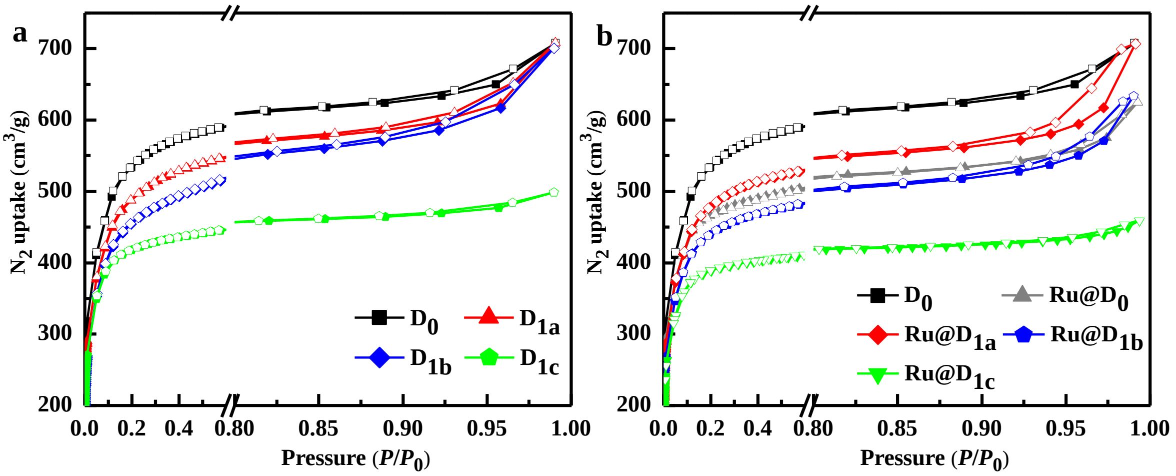

Supplementary Fig. 14 N_2 sorption isotherms at 78 K for D_0 and $D_{1a\text{-}c}$ (**a**), and that for D_0, Ru@D_0 and Ru@$D_{1a\text{-}c}$ (**b**) closed and open symbols represent the adsorption and desorption isotherms, respectively.

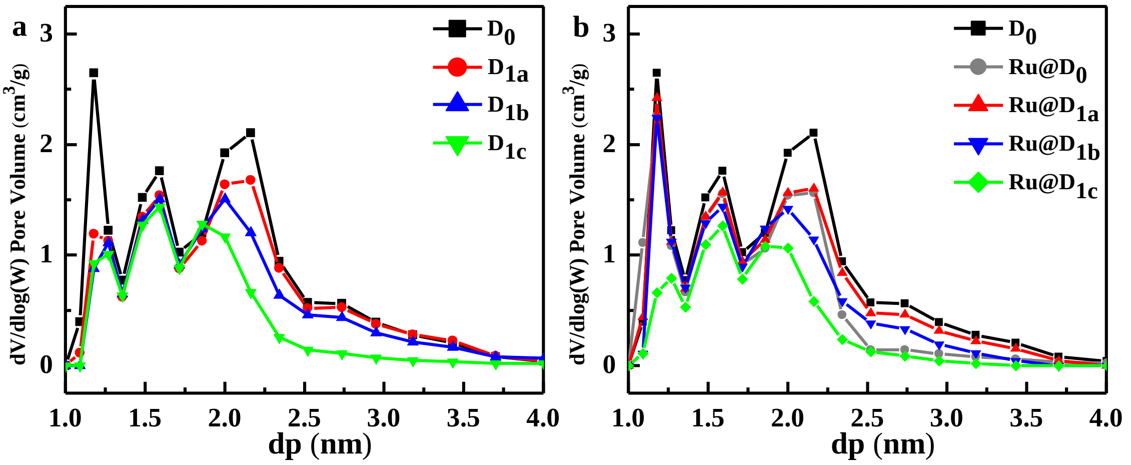

Supplementary Fig. 15 Pore size distributions of D_0 and $D_{1a\text{-}c}$ (**a**), and that of D_0, Ru@D_0 and Ru@$D_{1a\text{-}c}$ (**b**) calculated by DFT method.

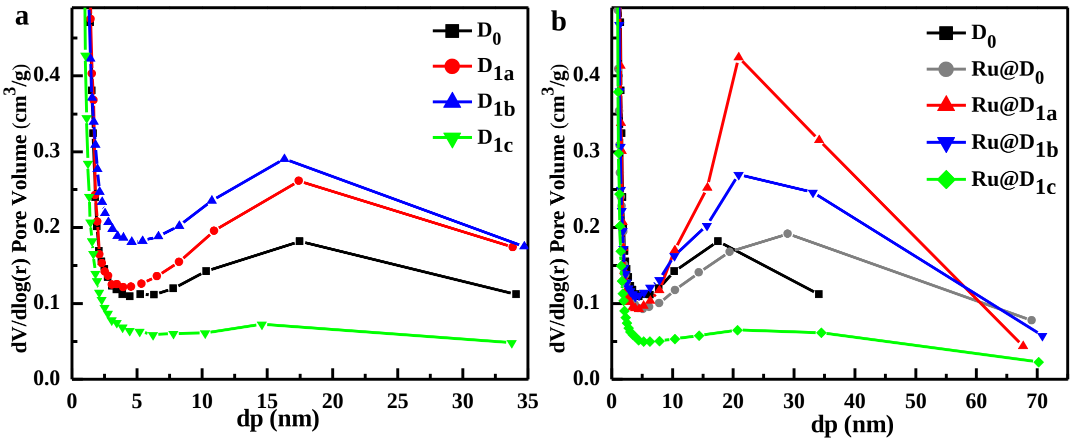

Supplementary Fig. 16 Pore size distributions of D_0 and $D_{1a\text{-}c}$ (**a**), and that of D_0, Ru@D_0 and Ru@$D_{1a\text{-}c}$ (**b**)

calculated by BJH method.

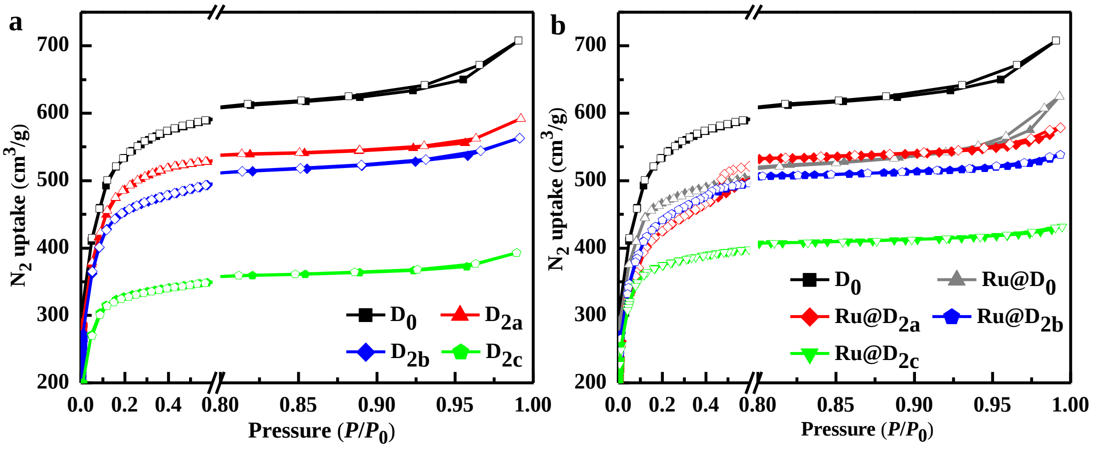

Supplementary Fig. 17 N_2 sorption isotherms at 78 K for D_0 and $D_{2a\text{-}c}$ (**a**), and that for D_0, $Ru@D_0$ and $Ru@D_{2a\text{-}c}$ (**b**) closed and open symbols represent the adsorption and desorption isotherms, respectively.

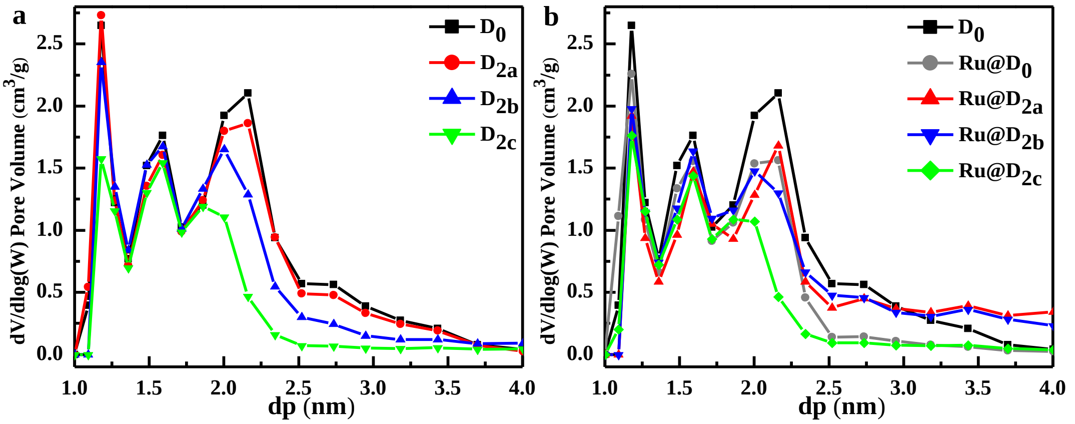

Supplementary Fig. 18 Pore size distributions of D_0 and $D_{2a\text{-}c}$ (**a**), and that of D_0, $Ru@D_0$ and $Ru@D_{2a\text{-}c}$ (**b**) calculated by DFT method.

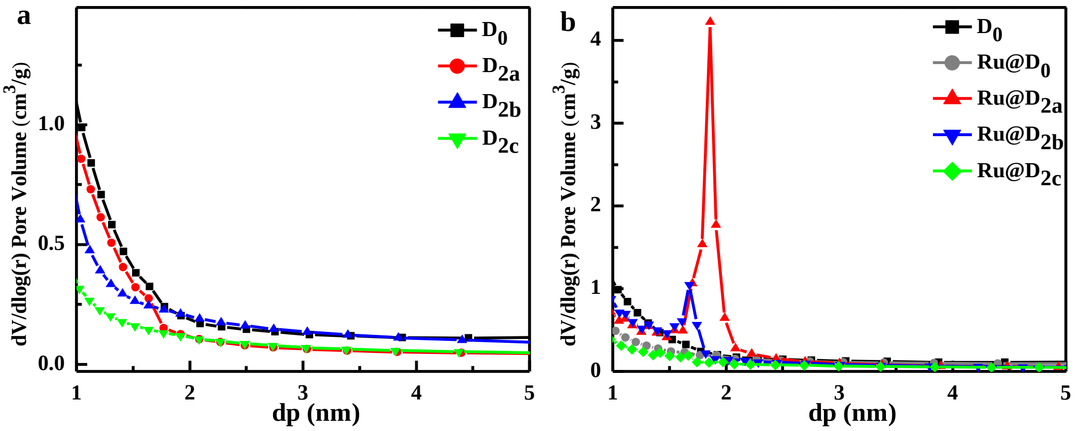

Supplementary Fig. 19 Pore size distributions of D_0 and $D_{2a\text{-}c}$ (**a**), and that of D_0, $Ru@D_0$ and $Ru@D_{2a\text{-}c}$ (**b**) calculated by BJH method.

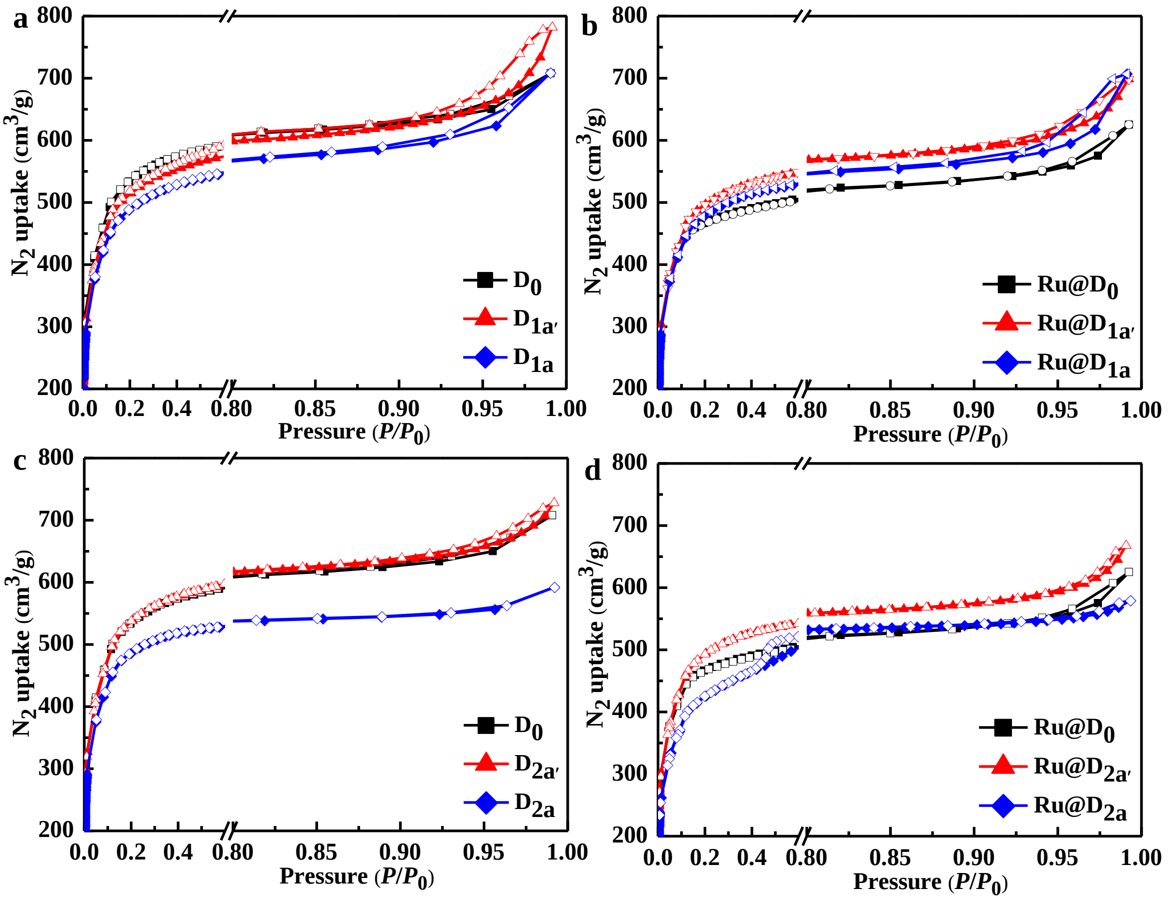

Supplementary Fig. 20 N_2 sorption isotherms at 78 K for D_0, $D_{1a'}$ and D_{1a} (**a**), Ru@D_0, Ru@$D_{1a'}$ and Ru@D_{1a} (**b**), D_0, $D_{2a'}$ and D_{2a} (**c**), Ru@D_0, Ru@$D_{2a'}$ and Ru@D_{2a} (**d**), closed and open symbols represent the adsorption and desorption isotherms, respectively.

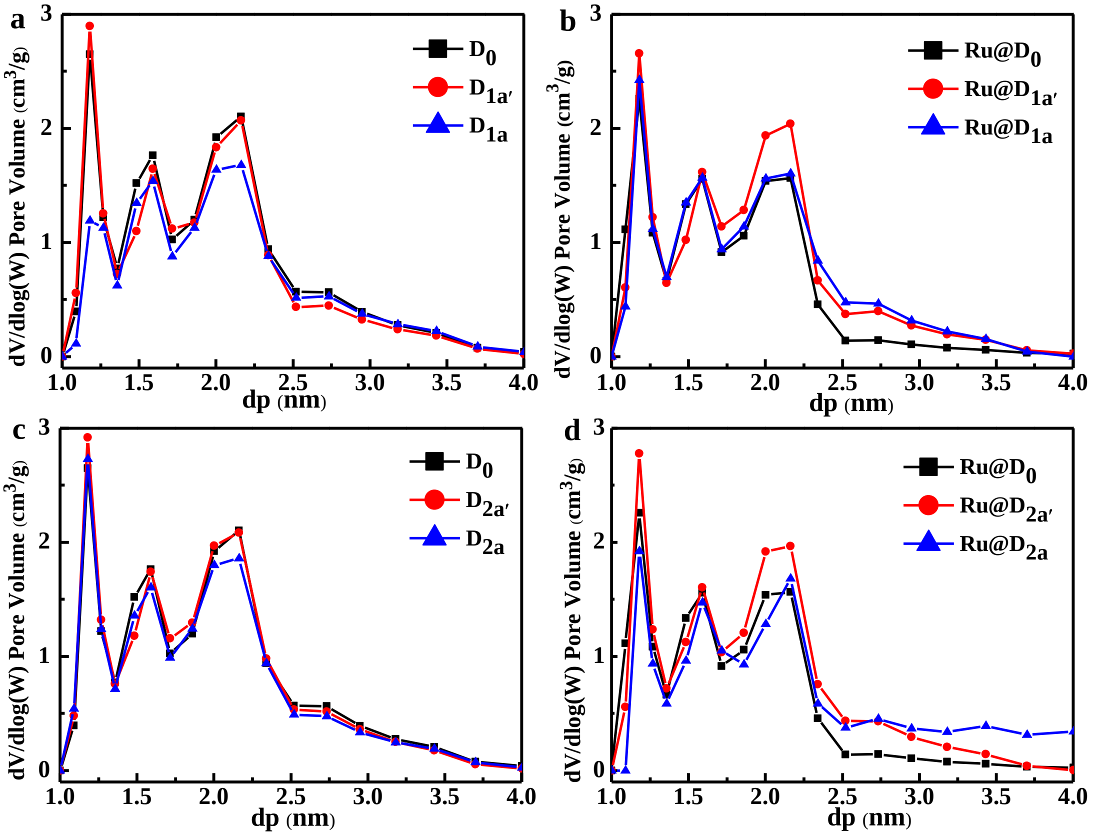

Supplementary Fig. 21 Pore size distributions of D_0, $D_{1a'}$ and D_{1a} (**a**), Ru@D_0, Ru@$D_{1a'}$ and Ru@D_{1a} (**b**), D_0, $D_{2a'}$ and D_{2a} (**c**), Ru@D_0, Ru@$D_{2a'}$ and Ru@D_{2a} (**d**) calculated by DFT method.

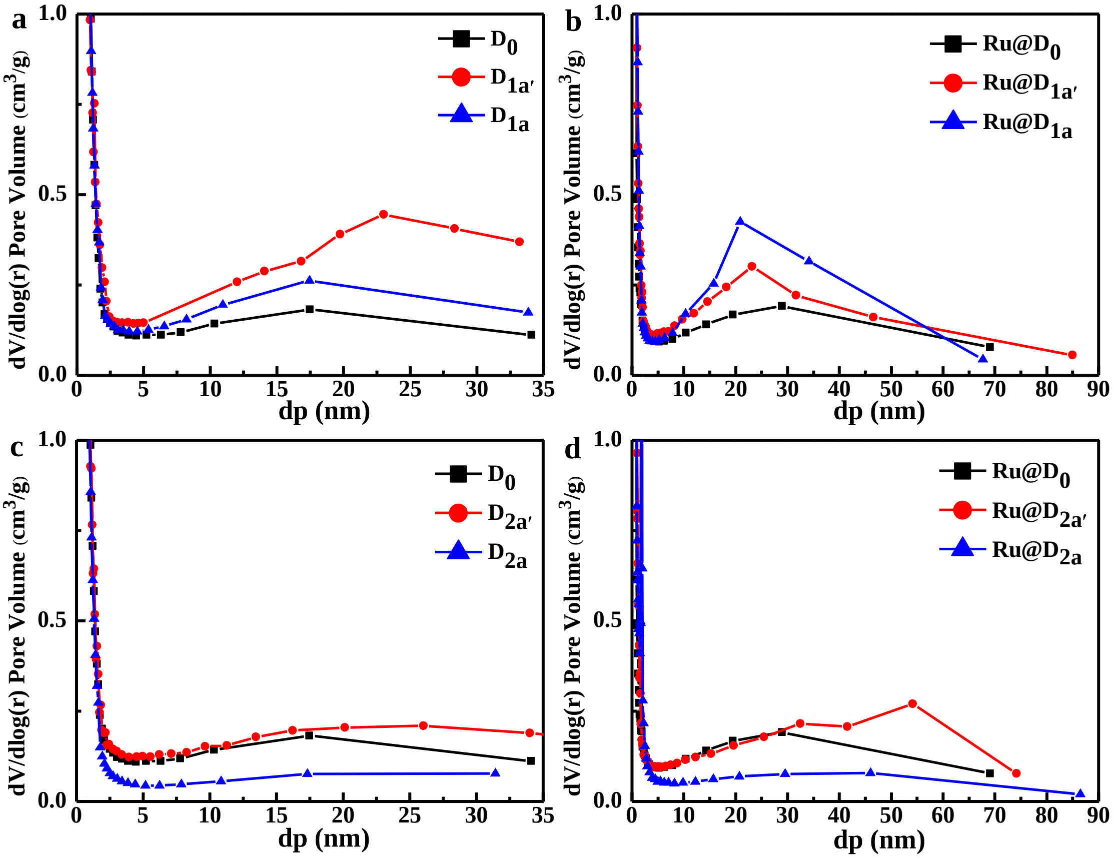

Supplementary Fig. 22 Pore size distributions of D_0, $D_{1a'}$ and D_{1a} (**a**), Ru@D_0, Ru@$D_{1a'}$ and Ru@D_{1a} (**b**), D_0, $D_{2a'}$ and D_{2a} (**c**), Ru@D_0, Ru@$D_{2a'}$ and Ru@D_{2a} (**d**) calculated by BJH method.

Supplementary Table 4 BET surface areas, Langmuir surface areas, and total pore volumes of dry, activated D_0, D_{1a-c}, D_{2a-c}, Ru@D_0, Ru@D_{1a-c} and Ru@D_{2a-c}. All the calculations are based on their N_2 adsorption isotherms at 78 K.

Samples	BET [m^2/g]	Langmuir [m^2/g]	Total pore volume ($P/$ = 0.95) [cm^3/g]	Types of physisorption isotherms
D_0	1738.487	2653.706	0.997	IV
$D_{1a'}$	1654.494	2705.093	1.016	IV
D_{1a}	1597.892	2512.536	0.943	IV
D_{1b}	1482.243	2426.355	0.932	IV
D_{1c}	1310.583	2007.825	0.734	I
$D_{2a'}$	1729.168	2747.437	1.035	IV
D_{2a}	1581.067	2342.204	0.855	I
D_{2b}	1486.431	2222.578	0.826	I
D_{2c}	1027.526	1558.522	0.572	I
Ru@D_0	1490.145	2336.233	0.859	IV
Ru@$D_{1a'}$	1594.473	2552.455	0.964	IV
Ru@D_{1a}	1561.366	2426.421	0.912	IV
Ru@D_{1b}	1418.836	2229.372	0.844	IV
Ru@D_{1c}	1190.563	1793.920	0.645	I
Ru@$D_{2a'}$	1576.974	2484.477	0.924	IV
Ru@D_{2a}	1404.548	2354.560	0.854	IV
Ru@D_{2b}	1444.196	2234.540	0.808	I
Ru@D_{2c}	1157.427	1800.007	0.649	I

N_2 gas adsorption/desorption has been conducted at 78 K to test the tolerance disparity of MIL-100-Cr to DL1 and DL2, to determine the different abilities to construct defects of DL1 and DL2, and to find out the framework evolutions caused by the incorporation of DL*x* and the Ru impregnation process. Importantly, the BET surface areas of all these samples remain high values (≥ 1027.526 m^2/g) (Supplementary Fig. 14-22, Supplementary Table 4), demonstrating the maintenance of the MIL-100-

Cr framework. The BET surface areas of $D_{1a\text{-}c}$, $D_{2a\text{-}c}$, Ru@$D_{1a\text{-}c}$ and Ru@$D_{2a\text{-}c}$ decrease along with incorporated DLx (x = 1, 2), being consistent with that of PCN-125 incorporated with a functionalized fragment of parent ligand TPTC[12]. The slight loss of BET surface areas of $D_{1a\text{-}b}$ and $D_{2a\text{-}b}$ along with a significant increase of mesopores in relation to that of D_0 demonstrates that $D_{1a\text{-}b}$ and $D_{2a\text{-}b}$ maintain the MIL-100-Cr framework with good crystallinity as the presence of mesopores is typically accompanied by a certain loss of specific surface areas for the modified MOFs[12-18]. In comparison with Ru@D_0 (1490.145 m^2g^{-1}), Ru@D_{1b} and Ru@$D_{2a\text{-}b}$ show comparable BET surface areas, ranging from 1404.548 to 1444.196 m^2g^{-1}, while Ru@$D_{1a'}$ (1594.473 m^2g^{-1}) and Ru@D_{1a} (1561.366 m^2g^{-1}) and Ru@$D_{2a'}$ (1576.974 m^2g^{-1}) show significantly higher BET surface areas and lower reduction of BET surface before and after loading Ru NPs. Moreover, mesopores are generated in Ru@$D_{1a\text{-}b}$ and Ru@$D_{2a\text{-}b}$. These results combined the PXRD, FTIR, TGA results of these samples confirm that Ru@DEMOFs with low feeding ratios of DL to TLs ($z \leqslant 30\%$) maintain well the framework of MIL-100-Cr with good crystallinity. The reduced BET surface areas of D_{xc} (x = 1, 2) and Ru@D_{xc} (x = 1, 2) compared to that of D_0 and Ru@D_0, respectively, don't result in the increase of mesopore, primarily attributed to a certain degree of blocking pores due to local disorder of these samples.

Supplementary Table 5 Ru loaded amount analyzed by ICP-OES.

Catalyst	Feeding ratio of Ru to MOF (wt%)	Lost mass percentage of MOFs supporters (%)	Loading ratio of Ru to MOF (wt%)	Maintaining ratio of Ru to MOF (wt%) after catalysis for 12 runs (wt%)	Ru residual content (%)
Ru@D_0	2.5	10.80	2.711	0.570	21.03
Ru@$D_{1a'}$	2.5	3.30	2.493	0.948	38.03
Ru@D_{1a}	2.5	3.40	2.421	1.036	42.81
Ru@D_{1b}	2.5	8.20	2.546	1.189	46.70
Ru@D_{1c}	2.5	23.20	3.044	1.322	43.43
Ru@$D_{2a'}$	2.5	5.50	2.502	1.097	43.84
Ru@D_{2a}	2.5	6.70	2.508	1.143	45.57
Ru@D_{2b}	2.5	9.70	2.589	1.430	55.23
Ru@D_{2c}	2.5	12.50	2.671	1.510	56.53

Supplementary Table 5 shows that the Ru loading amounts, obtained from the inductively coupled plasma optical emission spectrometry (ICP-OES), are increased significantly along with the increase of the ratio of incorporated DL*x* (*x* =1, 2) to TL for both kinds of Ru@DEMOFs. The slightly lower impregnated amounts of Ru NPs in Ru@$D_{1a'\text{-}b}$ before and after 12 runs of catalysis, compared with that of Ru@$D_{2a'\text{-}b}$, respectively, are mainly attributed to the lower incorporated amount of DL1 in Ru@$D_{1a'\text{-}b}$ in contrast by the incorporated amount of DL2 in Ru@$D_{2a'\text{-}b}$, respectively, and/or the higher stability of $D_{1a'\text{-}b}$ in relation to that of $D_{2a'\text{-}b}$, respectively, under Ru impregnation process, deduced from the lost mass percentage of each DEMOF after embedding Ru NPs and the leaching amount of chromium ions from the framework. The highest impregnated amount of Ru NPs in Ru@D_{1c} before catalysis is attributed to the lowest stability of D_{1c}, confirmed by the highest lost mass percentage of D_{1c} after embedding Ru NPs. The amounts of impregnated Ru NPs in both Ru@$D_{1a\text{-}c}$ and Ru@$D_{2a\text{-}c}$ decrease after 12 runs of catalysis, mainly attributed to the high-speed stirring of the reaction solution. The variety trends of the amount of Ru NPs for both two types of Ru@DEMOFs after 12 runs of catalysis are consistent as that before catalysis. And the higher contents of Ru NPs in Ru@D_{2c} after 12 runs of

catalysis in relation to that of Ru@D_{1c} is primarily due to the higher stability of Ru@D_{2c} than that of Ru@D_{1c}. After 12 runs of catalysis, the contents of Ru NPs in all Ru@DEMOFs are much higher than that of Ru@D_0, demonstrating that the defects can stabilize the impregnated Ru NPs.

1.12 STEM analysis

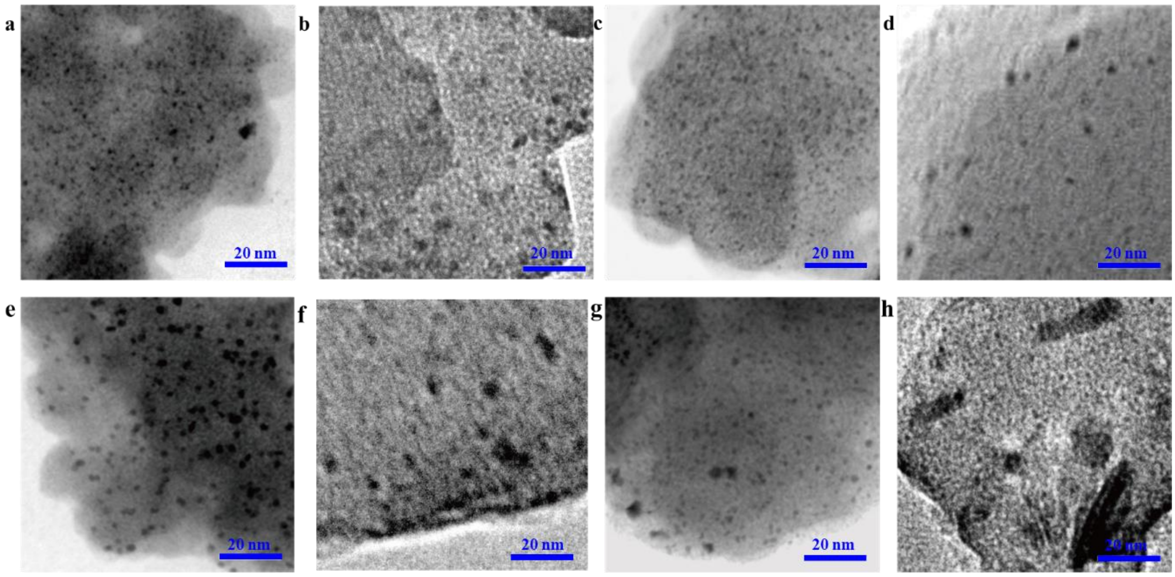

Supplementary Fig. 23 The STEM images of Ru@D_{1b} (**a**), Ru@D_{1c} (**b**), Ru@D_{2b} (**c**), and Ru@D_{2c} (**d**) before catalyzing biomass hydrogenation of D-glucose to sorbitol, and the STEM images of Ru@D_{1b} (**e**), Ru@D_{1c} (**f**), Ru@D_{2b} (**g**), and Ru@D_{2c} (**h**) after catalyzing biomass hydrogenation of D-glucose to sorbitol for 12 runs. Scale bars: 20 nm.

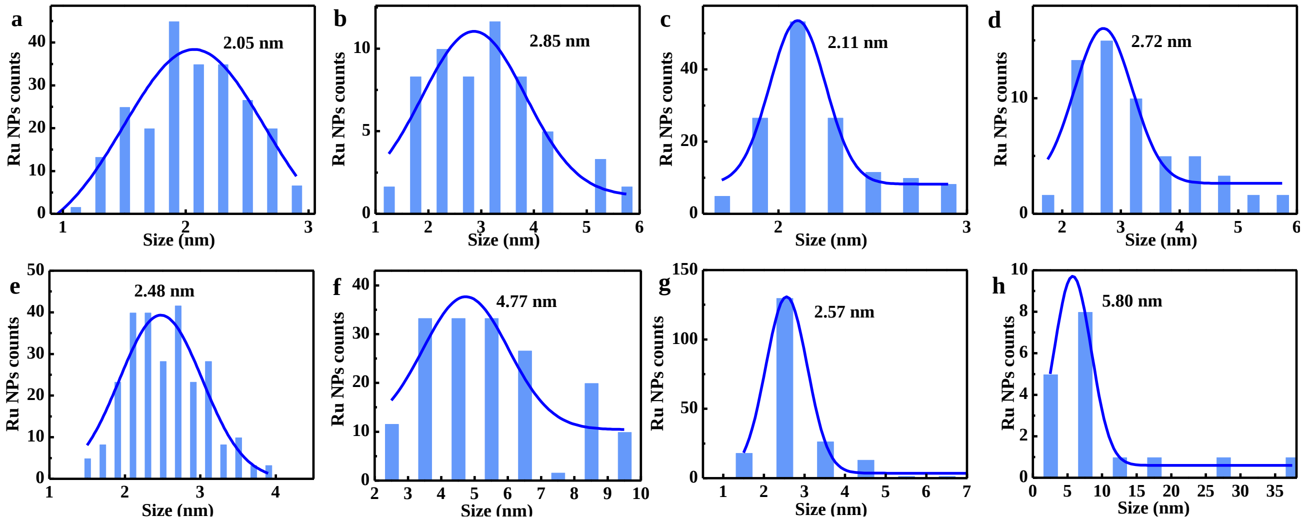

Supplementary Fig. 24 Particle size distributions of Ru NPs, determined by the STEM images, in Ru@D_{1b} (**a**), Ru@D_{1c} (**b**), Ru@D_{2b} (**c**), RuD_{2c} (**d**) before catalysis, and in Ru@D_{1b} (**e**), Ru@D_{1c} (**f**), Ru@D_{2b} (**g**), Ru@D_{2c} (**h**) after catalysis for 12 runs. Reaction conditions: D-glucose aqueous solution (25 wt%, 1.543 mol/L, 50 g), catalyst amount (1 g), H_2 (5.0 MPa), temperature (120 °C), and stirring rate (600 rpm).

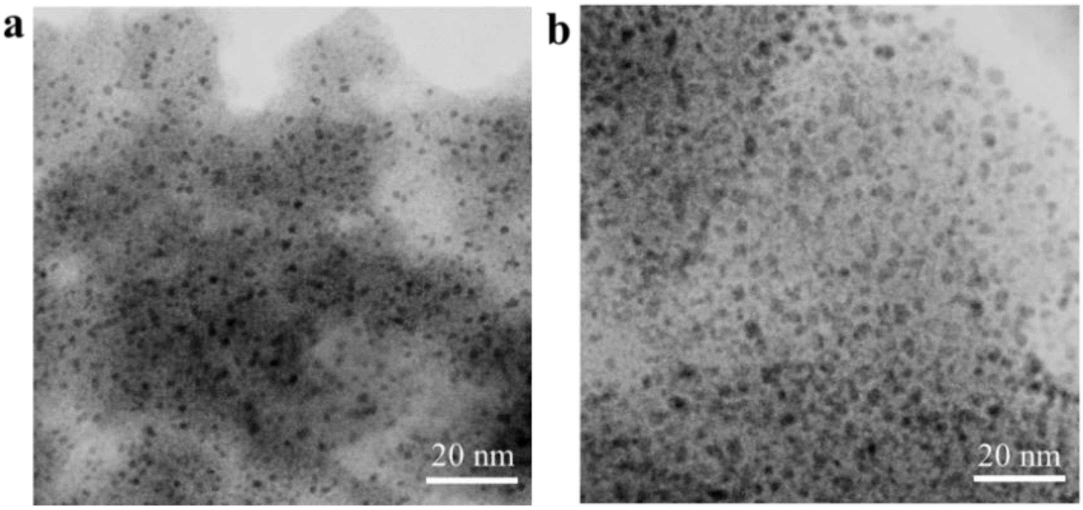

Supplementary Fig. 25. The STEM images of Ru@$D_{1a'}$ (**a**) and Ru@$D_{2a'}$ (**b**). Scale bars: 20 nm.

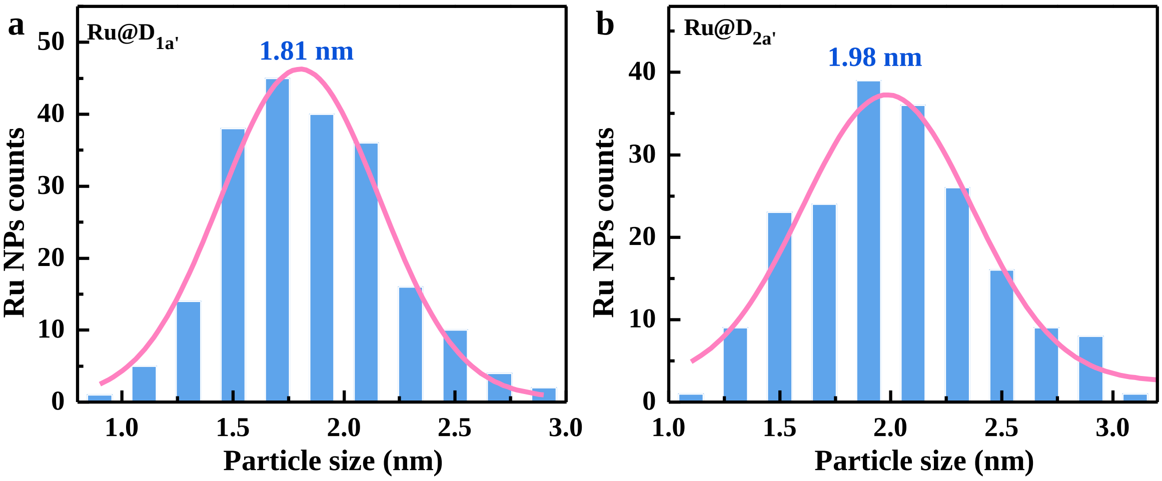

Supplementary Fig. 26 Particle size distributions of Ru NPs, decided from the STEM images, in Ru@$D_{1a'}$ (**a**), Ru@$D_{2a'}$ (**b**).

The STEM images (Supplementary Fig. 25) and the statistics of particle size distribution of Ru NPs (Supplementary Fig. 26) show that the size evolution of confined dominate Ru NPs in these DEMOFs with feeding ratio (*z*) ranging from 0% to 10% is consistent with that of Ru@DEMOFs with higher content of DL*x* (Supplementary Fig. 23, 24).

Supplementary Table 6 Comparison of the sizes of dominate Ru NPs in Ru@DEMOFs before and after 12 runs of hydrogenation reaction.

Catalyst	dominant Ru NPs diameter	dominant Ru NPs diameter	Diameters increase ratio (%) of

name	before 12 runs (nm)	after 12 runs (nm)	dominant Ru NPs
Ru@D_0	1.98	2.45	23.73
Ru@$D_{1a'}$	1.81	/	/
Ru@D_{1a}	1.95	2.20	12.83
Ru@D_{1b}	2.05	2.48	20.97
Ru@D_{1c}	2.85	4.77	67.36
Ru@$D_{2a'}$	1.98	/	/
Ru@D_{2a}	1.93	2.20	13.99
Ru@D_{2b}	2.11	2.57	21.80
Ru@D_{2c}	2.72	5.80	113.23

Particle size distributions of Ru NPs in all measured samples, determined by the STEM images, show that the diameters of dominant Ru NPs in both two kinds of Ru@DEMOFs increase along with the feeding ratio of DLx (x = 1, 2) to TL. These results illuminate that the Ru NPs sizes can be controllably adjusted by the introduced defects of different concentrations. After 12 runs of D-glucose selective hydrogenation, the size of dominant Ru NPs is enlarged (Fig. 2g-l; Supplementary Fig. 23, 24), and the higher concentration of defects leads to the larger aggregation degree of Ru NPs in these two types of Ru impregnated DEMOFs. However, the aggregation degree of Ru NPs in both Ru@$D_{1a\text{-}b}$ and Ru@$D_{2a\text{-}b}$ is lower than that in Ru@D_0 (Fig. 2g-i; Supplementary Tab. 6), demonstrating both types of defects of low concentration can stabilize Ru NPs. Furthermore, the aggregation degree of Ru NPs in Ru@$D_{1a\text{-}c}$ is lower than that in Ru@$D_{2a\text{-}c}$ with the same z of DLx, illuminating that the type-A defects can stabilize Ru NPs more efficiently than type-B defects against aggregation during catalytic reaction, mainly due to the stronger anchoring effect between confined Ru NPs and basic pyridyl-N atoms at type-A defects. The above results demonstrate that a rational tuning of defects can prevent the aggregation of Ru NPs embedded in Ru@DEMOFs.

1.13 CO pulse chemisorption measurements

Supplementary Table 7 Characteristics of Ru NPs active sites in the as-prepared catalysts obtained from CO chemisorption.

Catalyst name	Metallic surface area (m^2/g sample)	Metallic surface area (m^2/g metal)	Metal dispersion (%)	Active particle diameter (nm)
Ru@D_0	40.2528	139.2830	19.8766	5.786
Ru@$D_{1a'}$	41.4016	147.5992	20.0477	5.7448
Ru@D_{1a}	39.3619	137.5808	19.5389	5.888
Ru@D_{1b}	32.6371	113.5600	16.1627	7.123
Ru@D_{1c}	20.6634	70.6922	10.1431	11.361
Ru@$D_{2a'}$	42.3096	150.6215	21.1571	5.4449
Ru@D_{2a}	41.9960	146.3273	20.8125	5.542
Ru@D_{2b}	39.2377	136.3368	19.4174	5.925
Ru@D_{2c}	28.6363	99.2250	14.1507	8.154

The average diameters of Ru NPs in both two kinds of Ru@DEMOFs, obtained from CO pulse chemisorption measurements, also increase upon increasing the feeding ratio of DLx (x = 1, 2) to TL, being consistent with that obtained from STEM measurements. The dispersions of Ru NPs in both two kinds of Ru@DEMOFs decrease along with increasing the feeding ratio of DLx (x = 1, 2) to TL, primarily attributed to the aggregation of defects with high concentrations. These results further confirm that the sizes and distributions of Ru NPs can be controllably adjusted by the type of introduced defects as well as their concentrations.

1.14 CO adsorption UHV-FTIR spectra

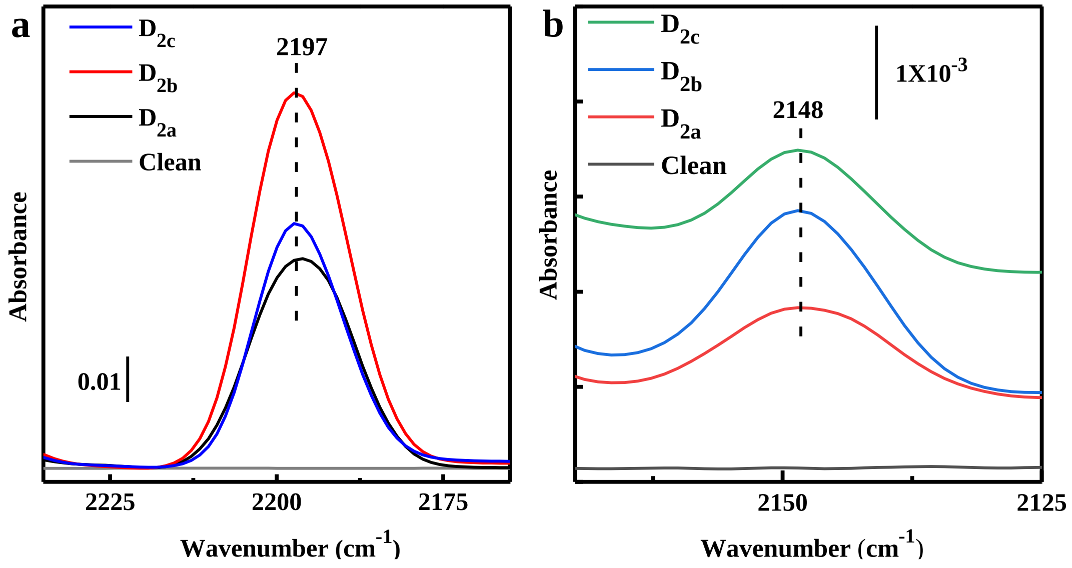

Supplementary Fig. 27 UHV-FTIR spectra obtained after CO adsorption (0.01 mbar) at 110 K on D_{2a-c} DEMOFs: (a) in the Cr^{3+}-related CO vibration region; (b) in the $Cr^{\delta+}$-related CO vibration region. Prior to exposure, each sample was heated to 500 K to remove all adsorbed species.

In relation to that of Ru@D_0, the main bands stemming from both CO-Cr^{3+} (Supplementary Fig. 27a) and CO-$Cr^{\delta+}$ (Supplementary Fig. 27b) in D_{2a-c} shift slightly to lower frequency with increasing *z* of DL2. These results demonstrate the formation of electron-enriched $Cr^{\delta+}$ defects via the partial reduction of pristine Cr^{3+}-CUSs along with the incorporation of DL2.

1.15 Temperature-dependent CO desorption UHV-FTIR spectra

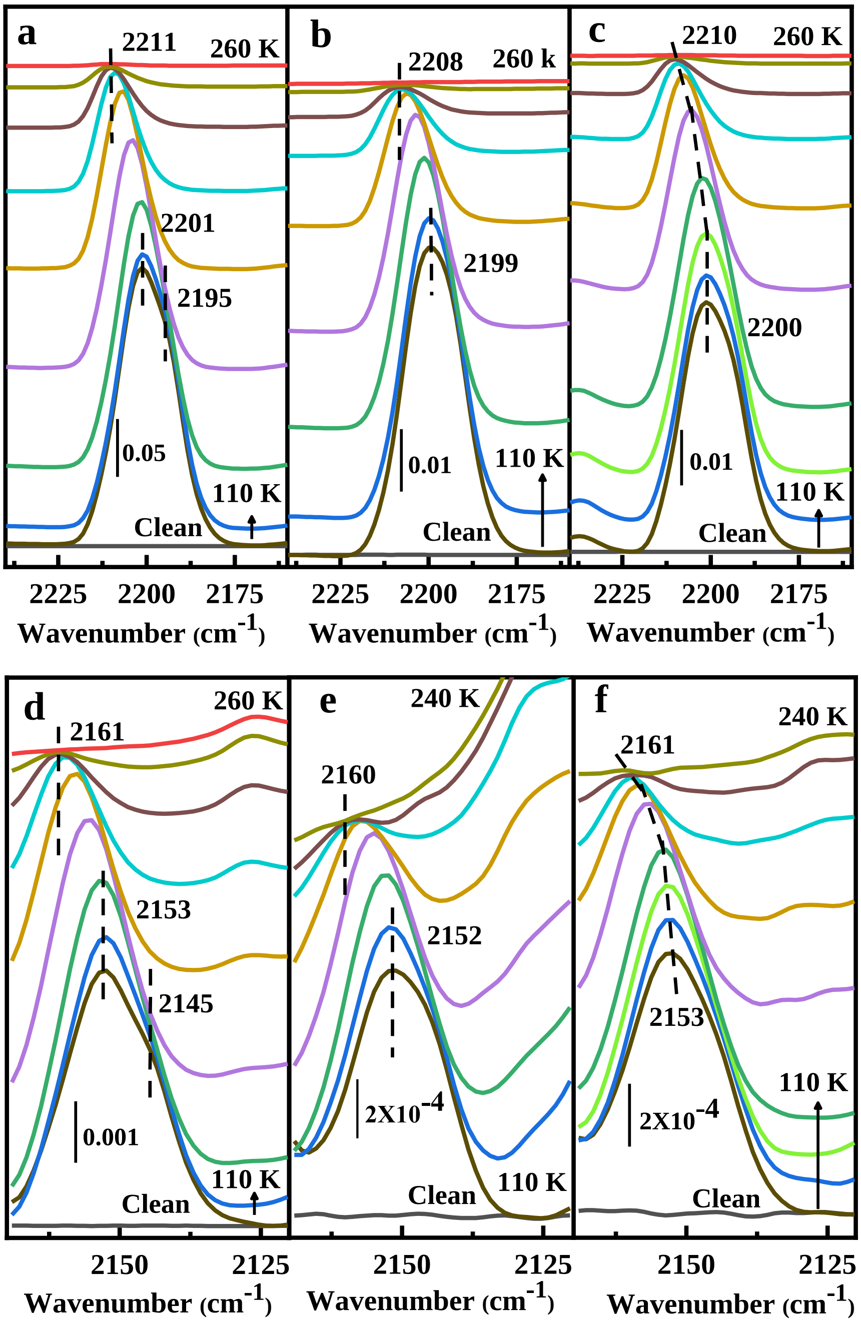

Supplementary Fig. 28 Thermal stability of various CO species adsorbed on accessible Cr-MSAS and impregnated Ru NPs, modified by varying incorporated DL*x* in type and concentration. Temperature-dependent UHV-FTIR spectra of CO on various Ru-impregnated MIL-100-Cr catalysts: (**a-c**) in the Cr^{3+}-related CO vibration region for Ru@D_0 (**a**), Ru@D_{1a} (**b**) and Ru@D_{2a} (**c**); (**d-f**) in the Cr^{3+}-related CO vibration region for Ru@D_0 (**d**), Ru@D_{1a} (**e**) and Ru@D_{2a} (**f**). All samples were exposed to CO (0.01 mbar) at ~110 K, and then heated to the indicated temperatures. Prior to exposure, each sample was heated to 500 K to remove all adsorbed species.

1.16 XPS analysis of Au@DEMOFs

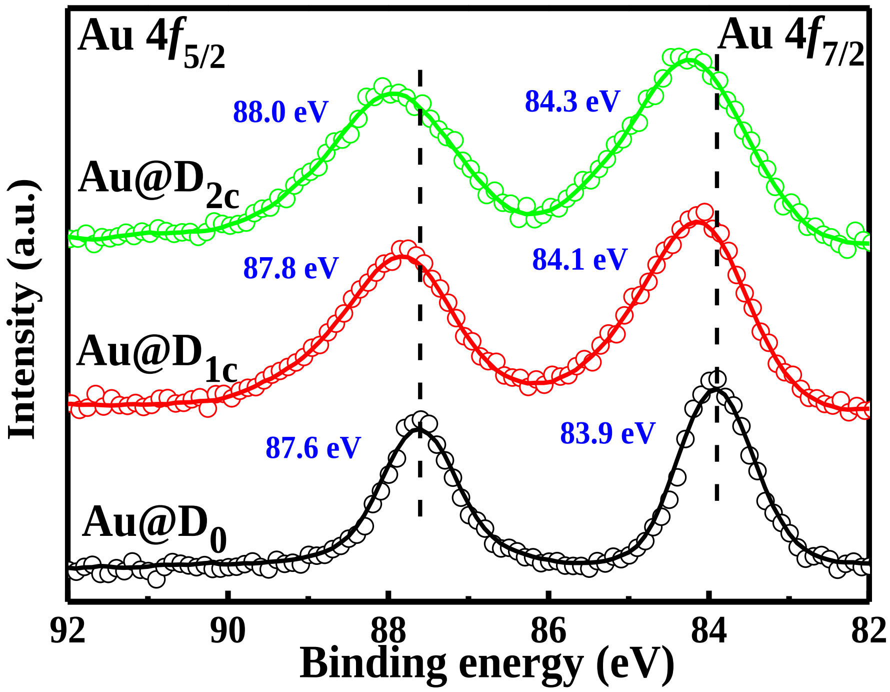

Supplementary Fig. 29 XPS spectra of Au $4f_{7/2}/4f_{5/2}$ doublet region obtained for Au@D_0, Au@D_{1c} and Au@D_{2c}.

The binding energy of the Ru 3*d* is very close to that of the more intense C 1*s* peaks in the XPS spectra. However, the binding energy of Au 4*f* is non-overlapping with all elements of the framework, and thus can be used as a solid reference for a reliable analysis of the electronic structure changes. Based on these considerations, to further validate the charge transfer between metal NPs and the framework of DEMOFs, we have performed additional experiments including the synthesis of Au-NPs impregnated D_0 (Au@D_0), D_{1c} (Au@D_{1c}) and D_{2c} (Au@D_{2c}) DEMOFs, as well as subsequent XPS characterization. Given that all Au impregnated MOF catalysts Au@D_0, Au@$D_{1a\text{-}c}$ and Au@$D_{2a\text{-}c}$ were obtained at the same reduction condition (under 4 MPa hydrogen pressure at 120 ºC for 180 min) in a stainless autoclave, the presence of oxidized Au species can be excluded. The diameters of dominant Ru NPs in both two kinds of Ru@DEMOFs increase along with the feeding ratio (z) of DLx (x = 1, 2) to TL, and that of these two kinds of Ru@DEMOFs with same z is comparable (Supplementary Table 6). Generally, the larger size of metal nanoparticles results in the smaller binding energy. On consideration of these two aspects, the binding energy of Au@D_{1c} is expected to be comparable to that of Au@D_{2c}, and both of which should be smaller than that of Au@D_0. However, the XPS spectra (Supplementary Fig. 29) show that the binding energies of the Au $4f_{7/2}/4f_{5/2}$ doublet for both of the

Au@D_{1c} (84.1/87.8 eV) and Au@D_{2c} (84.3/88.0 eV) are higher than that of Au@D_0 (83.9/87.6 eV), revealing that the embedded Au NPs in D_{1c} and D_{2c} DEMOFs are slightly positively charged. This finding is attributed to the electronic interaction between the embedded Au NPs and defective $Cr^{\delta+}$-CUSs ($\delta < 3$) acting as Lewis acid sites (electron acceptors) that lose one coordinating carboxyl in DEMOFs (see Fig. 2a-b). Furthermore, the binding energy of the Au $4f_{7/2}/4f_{5/2}$ doublet in Au@D_{1c} is lower than that of Au@D_{2c}. indicating the additional electronic interaction between Au NPs and pyridyl-N atoms of DL1 in Au@D_{1c} as Lewis base sites (electron donors) that are absent for the Au@D_{2c} DEMOF. Overall, the above results confirm that the degree of charge transfer from metal NPs to the framework with type-B defects is larger than that to the framework containing type-A defects, and both of them are higher than that to the pristine framework.

1.17 IR - spectroscopy data of DEMOFs and G-DEMOFs

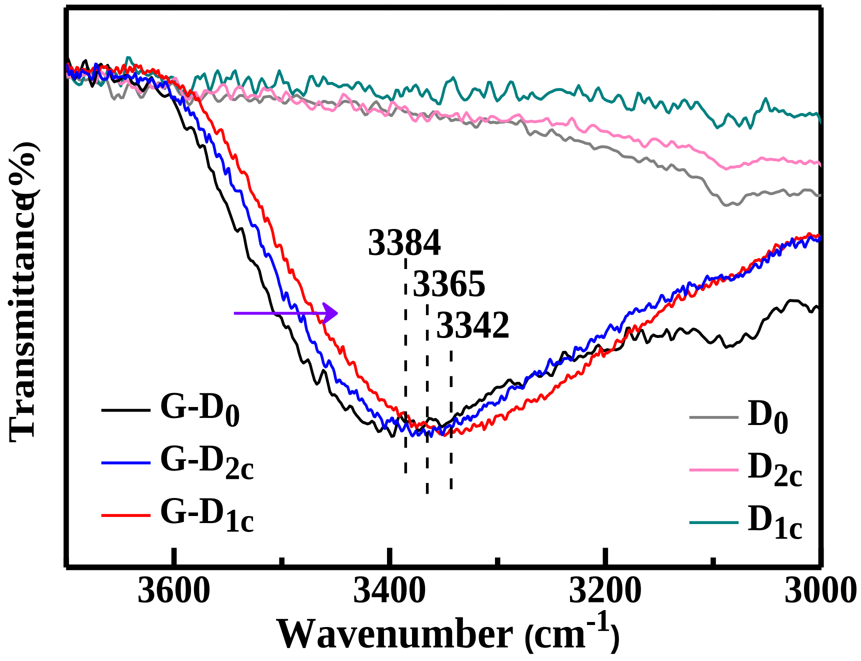

Supplementary Fig. 30 Routine FTIR spectra of the D-glucose impregnated G-D_0, G-D_{1c} and G-D_{2c}, in comparison with the activated unloaded D_0, D_{1c} and @D_{2c} samples.

Supplementary Fig. 30 shows that the strong broad band peaked at 3384 cm^{-1} and the weak band peaked at ~3081 and ~2932 cm^{-1} of D-glucose in G-D_0 shift to lower frequency and become weaker, respectively, in the FTIR spectra of G-D_{1c} and G-D_{2c}, demonstrating the presence of weak coordination bonds between D-glucose and defective MSAS. The lower frequency of the broad band covering 3600 to 3200 cm^{-1} of G-D_{1c} in relation to that of G-D_{2c} illustrates the formation of hydrogen bonds between

hydroxyl group in glucose molecule and N atoms of DL1 incorporated into the framework of D_{1c}. The disparity of the bands between the G-DEMOFs and G-Ru@DEMOFs confirms that Ru NPs impregnation process results in the evolution of both types of defects. These results validate our proposed synergistic catalytic mechanism of D-glucose selective hydrogenation to sorbitol for these two different kinds of Ru NPs impregnated DEMOFs (Fig. 1).

1.18 SEM analysis of Ru@DEMOFs

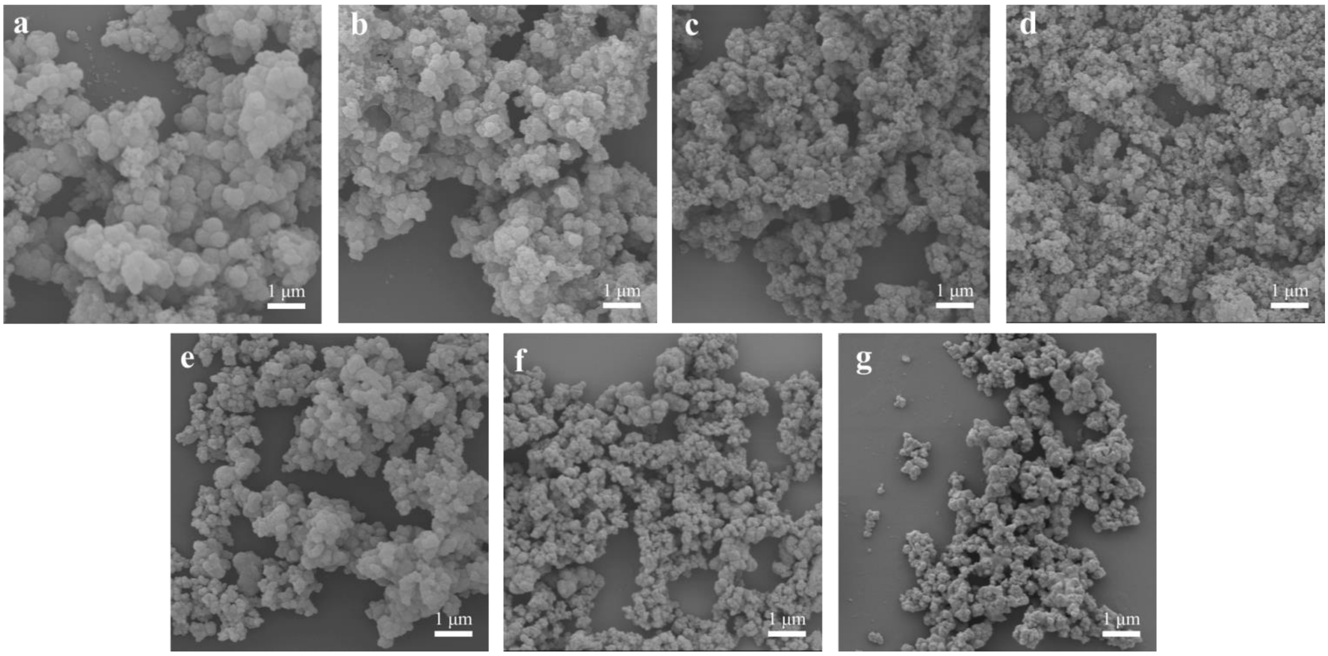

Supplementary Fig. 31 The SEM images of Ru@D_0 (**a**), Ru@D_{1a} (**b**), Ru@D_{1b} (**c**), and Ru@D_{1c} (**d**) Ru@D_{2a} (**e**), Ru@D_{2b} (**f**), Ru@D_{2c} (**g**), Scale bars: 1 µm.

As shown in Supplementary Fig. 31, the SEM images of all Ru@DEMOFs demonstrate the decrease of particle size along with increasing the feeding ratios (z) of DLx to TL (x =1, 2, z =10%, 30%, 50%). It is a main reason that the fine peaks in the PXRD of DEMOFs and Ru@DEMOFs gradually disappeared and merged into broad bands along with increasing the feeding ratio (z) of DLx to TL (x = 1, $z \geq 30\%$; $x = 2$, $z \geq 50\%$).

2. Catalysis test of D-glucose selective hydrogenation

2.1 Optimize catalytic conditions of Ru@D_0, Ru@D_{1a}, Ru@D_{2a} toward D-glucose selective hydrogenation.

2.1.1 Find out the optimal impregnated content of Ru in D_0, D_{1a} and D_{2a}.

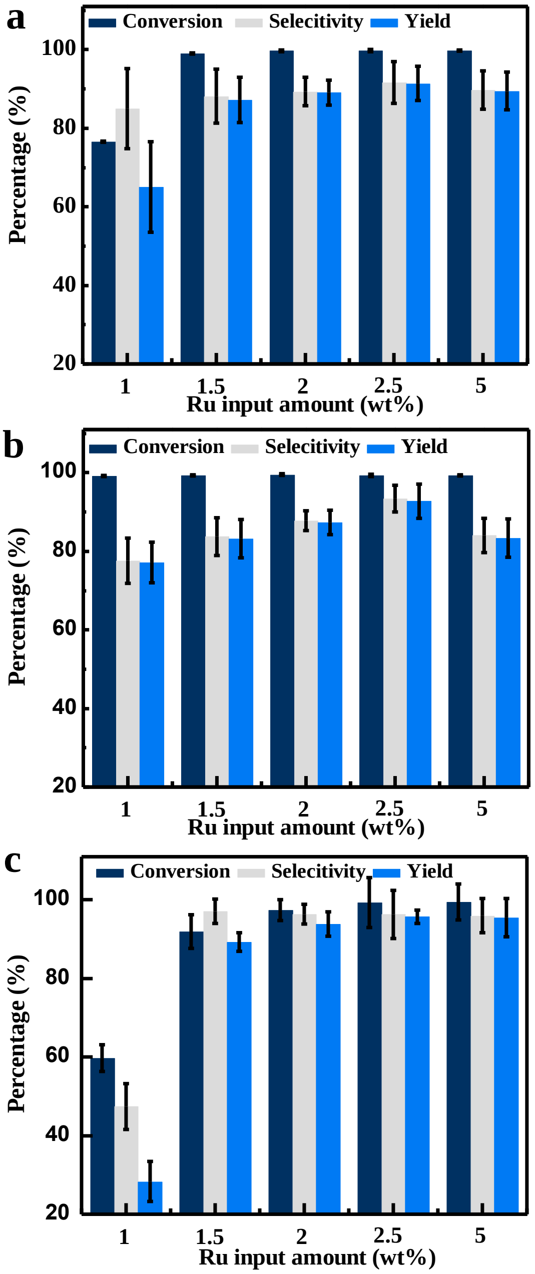

Supplementary Fig. 32 The conversions of D-glucose, selectivity and yields toward sorbitol for the D-glucose selective hydrogenation reactions catalyzed by Ru NPs impregnated D_0 (**a**), D_{1a} (**b**) and D_{2a} (**c**) with different loading amount ranging from 1 to 5 wt%. Reaction conditions: D-glucose aqueous solution (25 wt%, 1.543 mol/L, 50 g), catalyst (1 g), H_2 (5.0 MPa), temperature (120 °C), reaction time (150 min) and stirring rate (800 rpm).

The impregnated amount of Ru NPs plays a significant role in catalysis of D-glucose selective hydrogenation to sorbitol, consequently, the catalytic performances of Ru NPs impregnated D_0, D_{1a} and D_{2a} with different loading amounts of Ru element ranging from 1 to 5 wt% towards the D-glucose selective hydrogenation, have been investigated with all the other reaction conditions being fixed. As shown in Supplementary Fig. 32, the yields of sorbitol reach the maximum values when D_0, D_{1a} and D_{2a} were impregnated by $RuCl_3$ precursor containing 2.5 wt% Ru element, named as Ru@D_0, Ru@D_{1a} and Ru@D_{2a}, demonstrating that the optimal impregnated content of Ru NPs of these MOFs supporters is 2.5 wt%. Consequently, all the other Ru@DEMOFs catalysts were impregnated with Ru NPs by using $RuCl_3$ precursor containing 2.5 wt% Ru element.

2.1.2 Find out the optimal reaction temperature of Ru@D_0, Ru@D_{1a} and Ru@D_{2a}

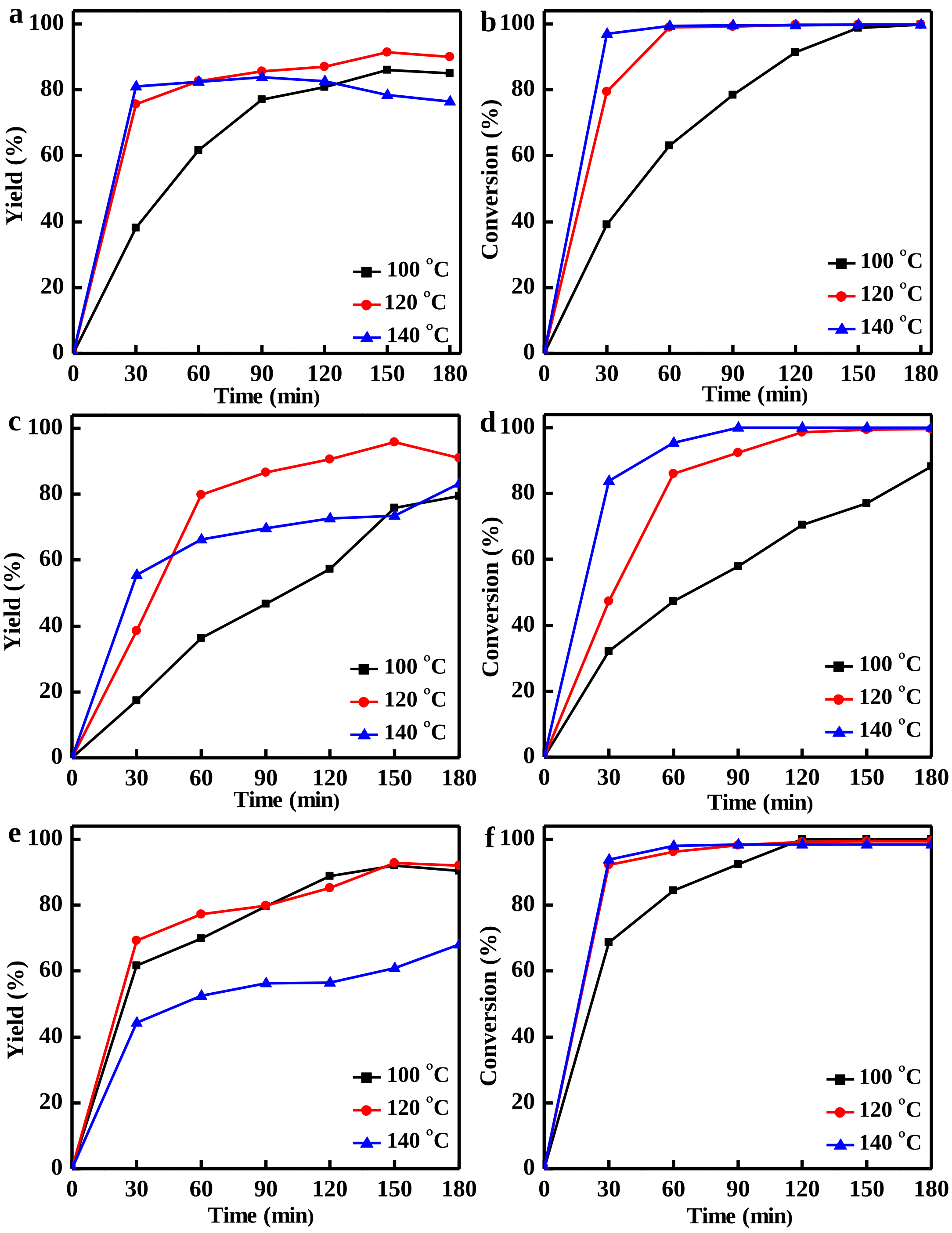

Supplementary Fig. 33 The curves of time-dependent yields of sorbitol (**a**, **c**, **e**) and conversions of D-glucose (**b**, **d**, **f**) toward the selective hydrogenation to sorbitol reactions catalyzed by Ru@D_0 (**a**, **b**), Ru@D_{1a} (**c**, **d**) and Ru@D_{2a} (**e**, **f**) at 100, 120 and 140 °C, respectively, under the same reaction conditions: D-glucose aqueous solution (25 wt%, 1.543 mol/L, 50 g), catalyst (1 g), H_2 (5.0 MPa), reaction time (180 min) and stirring rate (800 rpm).

As shown in Supplementary Fig. 33, the time-dependent catalytic performances of Ru@D_0, Ru@D_{1a}

and Ru@D_{2a} toward the D-glucose selective hydrogenation reaction at 100, 120 and 140 ºC, respectively, illuminate that the yields of sorbitol could reach the maximum values 91.52%, 96.01%, 92.82% for Ru@D_0, Ru@D_{1a} and Ru@D_{2a} at 120 ºC, respectively, when all the other reaction conditions were fixed. Consequently, to test the recyclability of all Ru@MOFs catalysts, all reactions were conducted at 120 ºC. These results demonstrate that both type-A and type-B defects can improve the catalytic performances of Ru impregnated MIL-100-Cr towards D-glucose selective hydrogenation to sorbitol, moreover, type-A defects show a better effect on the improvement of the catalytic performances for Ru impregnated MIL-100-Cr.

2.1.3 Find out the optimal reaction pressure of Ru@D_0, Ru@D_{1a} and Ru@D_{2a}

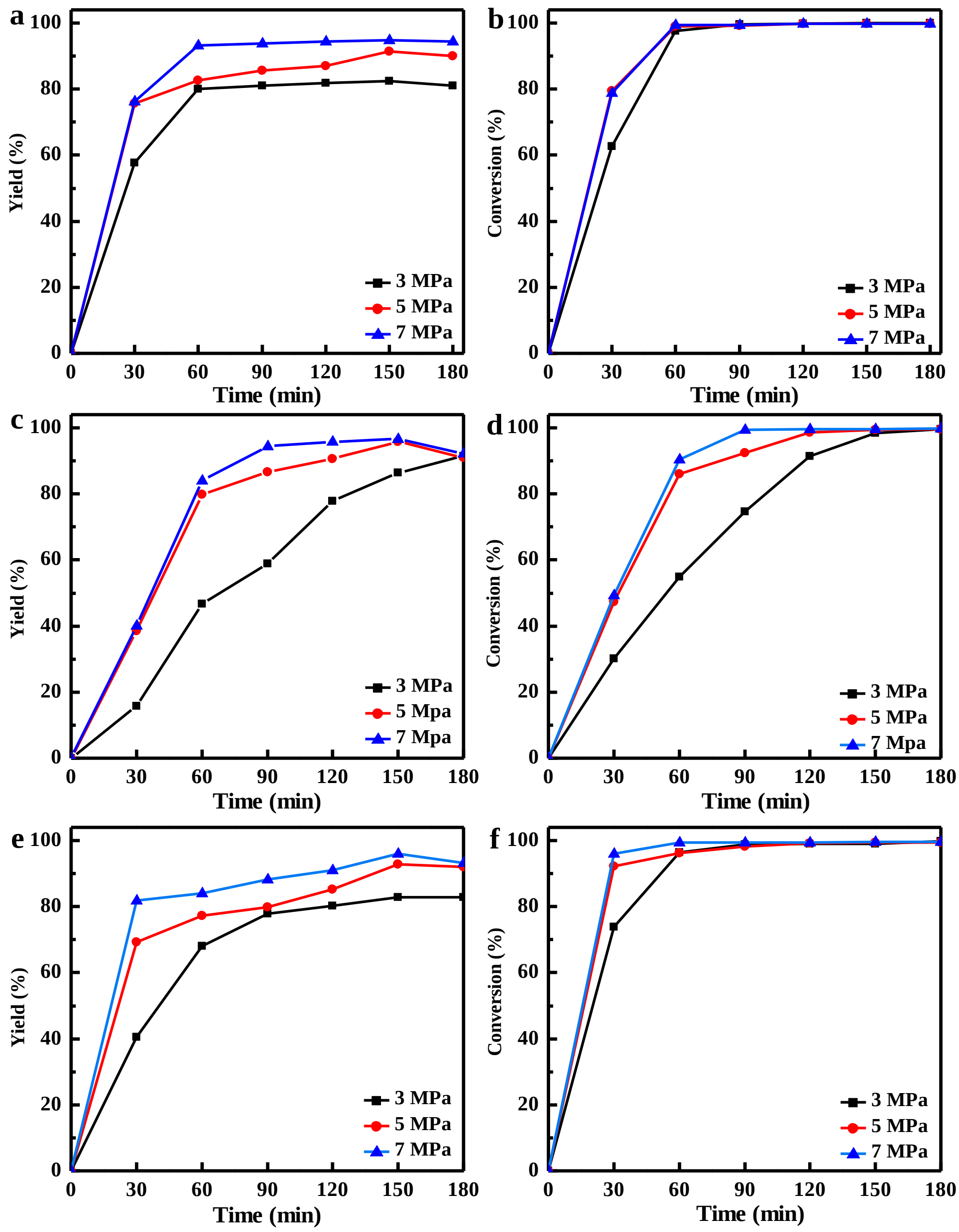

Supplementary Fig. 34 The curves of time-dependent yields of sorbitol (**a**, **c**, **e**) and conversions of D-glucose (**b**, **d**, **f**) toward the selective hydrogenation to sorbitol reactions catalyzed by Ru@D_0 (**a**, **b**), Ru@D_{1a} (**c**, **d**) and Ru@D_{2a} (**e**, **f**) at 3, 5 and 7 MPa, respectively, under the same reaction conditions: D-glucose aqueous solution (25 wt%, 1.543 mol/L, 50 g), catalyst (1 g), temperature (120 °C), reaction time (180 min) and stirring rate (800 rpm).

The sorbitol yields can be enhanced upon increasing the applying reaction pressure when all the other reaction conditions were fixed. However, considering the operation safety, economy and the tolerance of synthesized catalysts, all catalytic reactions in this work were conducted under the hydrogen pressure of 5 MPa.

2.1.4 Find out the optimal stirring rate of Ru@D_0, Ru@D_{1a} and Ru@D_{2a}

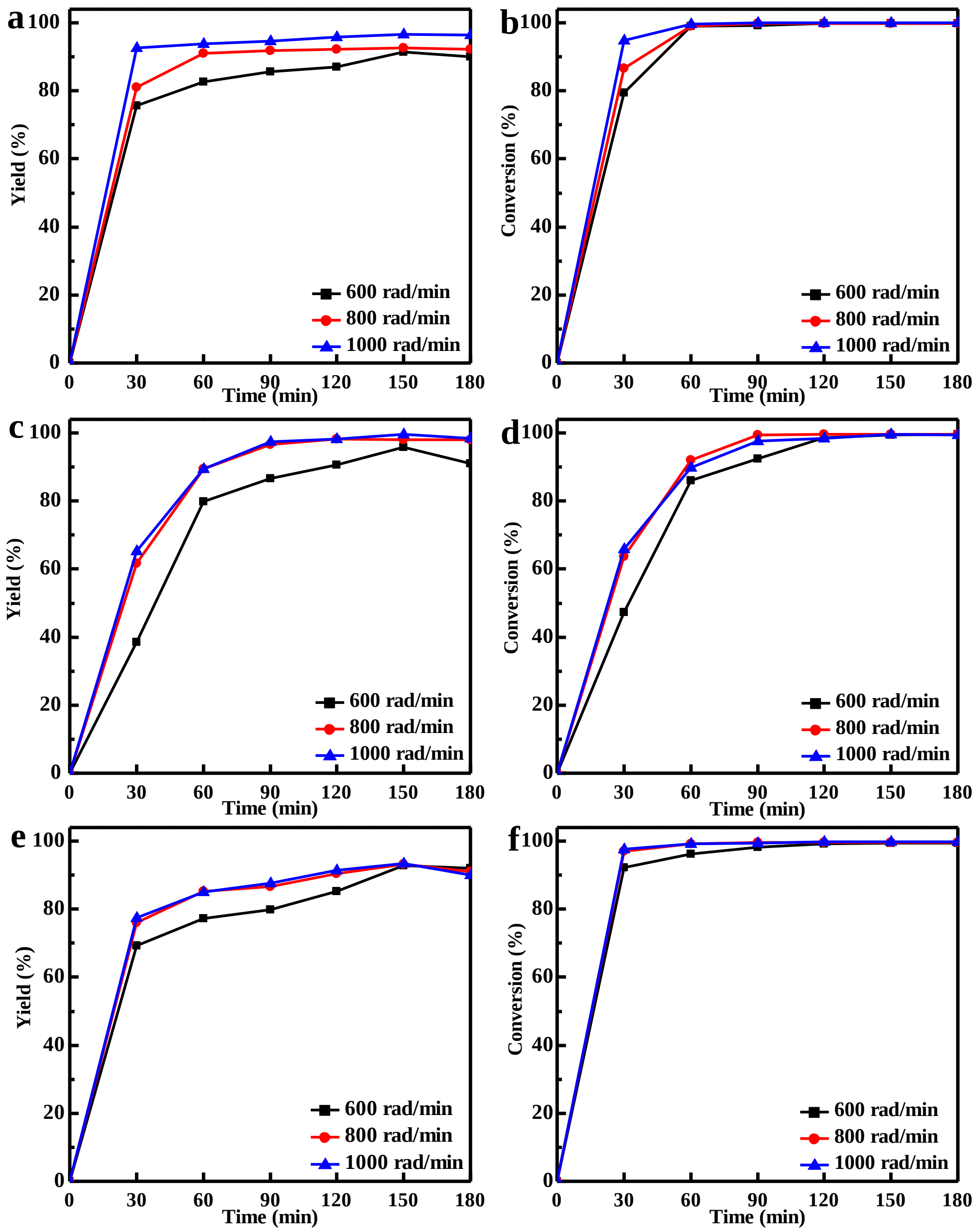

Supplementary Fig. 35 The curves of time-dependent yields of sorbitol (**a**, **c**, **e**) and conversions of D-glucose (**b**, **d**, **f**) toward selective hydrogenation to sorbitol reaction catalyzed by Ru@D_0 (**a**, **b**), Ru@D_{1a} (**c**, **d**) and Ru@D_{2a} (**e**, **f**) at 600, 800 and 1000 rpm, respectively under the same reaction conditions: D-glucose aqueous solution (25 wt%, 1.543 mol/L, 50 g), catalyst (1 g), hydrogen pressure (5 MPa), temperature (120 ºC), reaction time (180 min).

The maximum sorbitol yields of the selected investigated catalysts Ru@D_0, Ru@D_{1a} and Ru@D_{2a} can be raised along with the increase of applied stirring rate when all the other reaction conditions were fixed. However, considering the tolerance of these catalysts, the operation safety and economy, all catalytic reactions in this work were conducted at 800 rpm.

2.1.5 Find out the optimal reaction times of Ru@D$_0$, Ru@D$_{1a-c}$ and Ru@D$_{2a-c}$

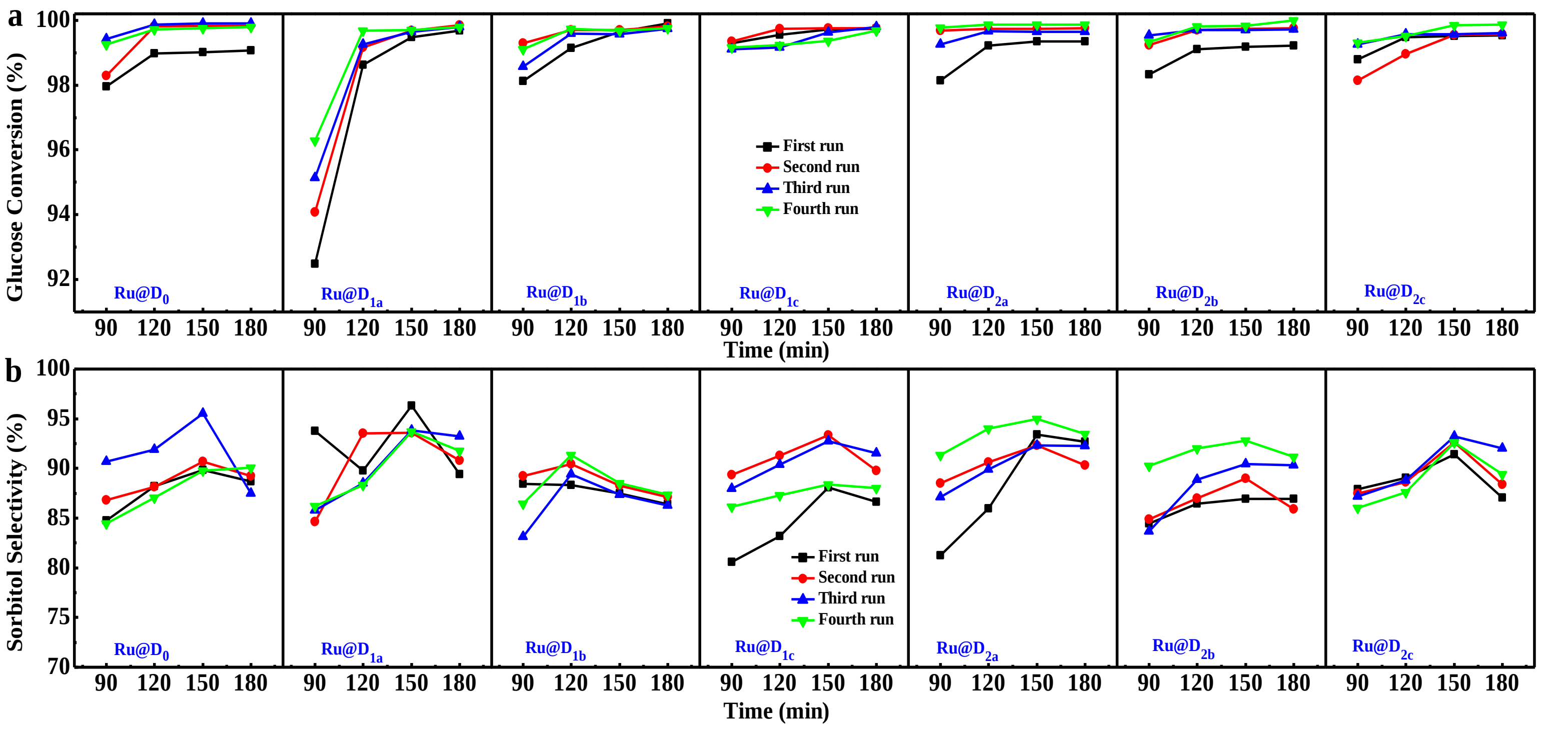

Supplementary Fig. 36 The curves of time-dependent D-glucose conversions (**a**) and selectivity to sorbitol (**b**) for the first four cycles of the reactions catalyzed by Ru@D$_0$, Ru@D$_{1a-c}$ and Ru@D$_{2a-c}$, respectively. Reaction conditions: D-glucose aqueous solution (25 wt%, 1.543 mol/L, 50 g), catalysts (1 g), hydrogen pressure (5 MPa), temperature (120 ºC) and stirring rate (800 rpm).

Supplementary Fig. 36 shows the curves of time-dependent conversion of D-glucose and selectivity to sorbitol for the first four cycles of the reactions catalyzed by Ru@D$_0$, Ru@D$_{1a-c}$ and Ru@D$_{2a-c}$ under the same optimized reaction conditions. The conversion of D-glucose and sorbitol selectivity for all these catalysts, except D$_{1b}$ obtaining the maximum sorbitol selectivity at 120 minutes, achieve the highest values after reacting for 150 minutes.

2.2 Blank Control Experiments

Supplementary Table 8 Conversion of D-glucose and selectivity to fructose and sorbitol toward D-glucose selective hydrogenation reactions without catalyst, and that catalyzed by pristine and defect engineered MIL-100-Cr, respectively.

**Samples**	**Activity/%**	**Fructose Selectivity/%**	**Sorbitol Selectivity/%**
Blank	4.228	16.911	57.450
D_0	8.151	20.010	20.856
D_{1a}	7.691	39.904	11.819
D_{1b}	8.052	52.186	8.135
D_{1c}	25.336	50.900	3.840
D_{2a}	8.876	30.002	14.973
D_{2b}	11.768	37.220	10.087
D_{2c}	23.135	59.127	3.492
Ru NPs	43.634	18.201	54.604

Reaction conditions: D-glucose aqueous solution (25 wt%, 1.543 mol/L, 50 g), catalyst (1 g), hydrogen pressure (5 MPa), temperature (120 °C), reaction time (180 min) and stirring rate (800 rpm).

In order to gain an in-depth understanding of the reaction mechanism based on the role of the MIL-100-Cr MOF supporters, artificially implanted defects and impregnated Ru NPs as well as their synergetic catalytic effect on D-glucose selective hydrogenation to sorbitol, the catalytic performances of Ru NPs, pristine and defect engineered MIL-100-Cr supporters have also been investigated. The detailed discussions have been given in the section "Determination of the roles of each active species and their synergistic catalytic mechanism" of the main text.

2.3 Kinetics Investigations

2.3.1 Determination of the reaction order of D-glucose selective hydrogenation to sorbitol.

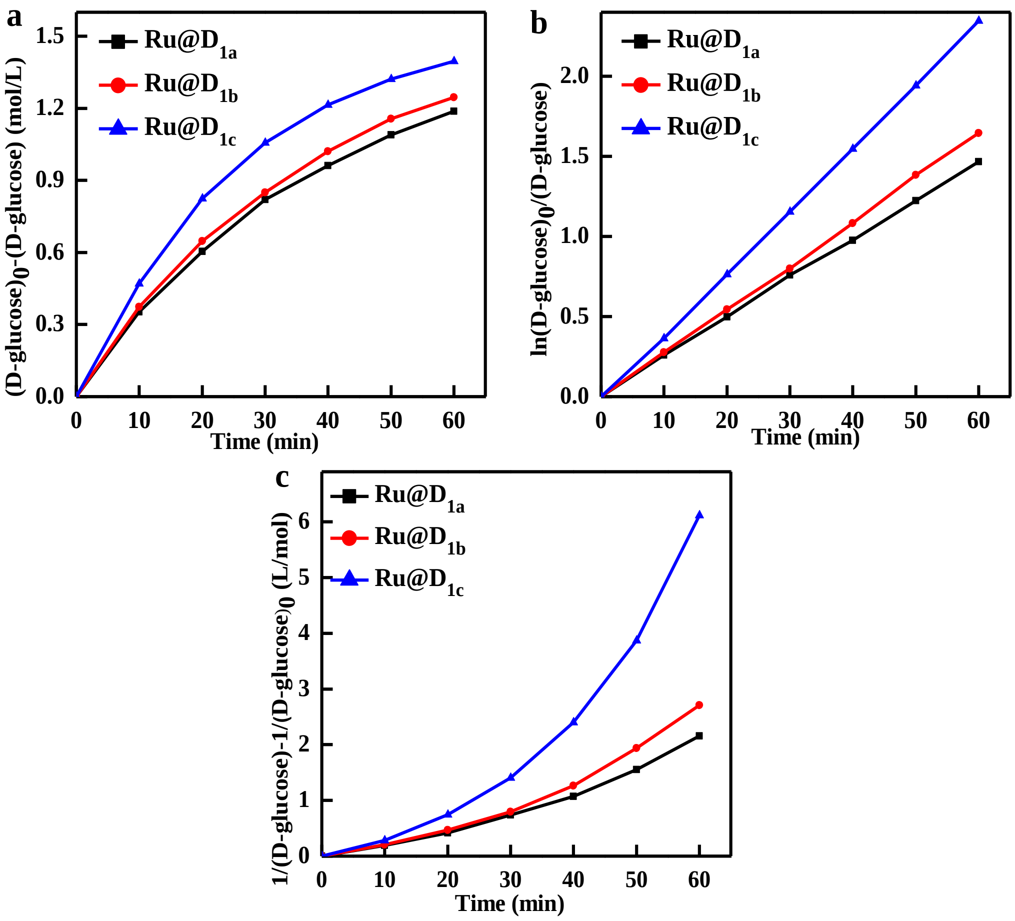

Supplementary Fig. 37 Test D-glucose selective hydrogenation to sorbitol in presence of Ru@D_0 and Ru@$D_{1a\text{-}c}$, respectively, as the zero-order reaction (**a**), the first-order reaction (**b**) and the second-order reaction (**c**). Reaction conditions: D-glucose aqueous solution (25 wt%, 1.543 mol/L, 50 g), catalyst (1 g), H_2 (5.0 MPa), temperature (120ºC) and stirring rate (800 rpm).

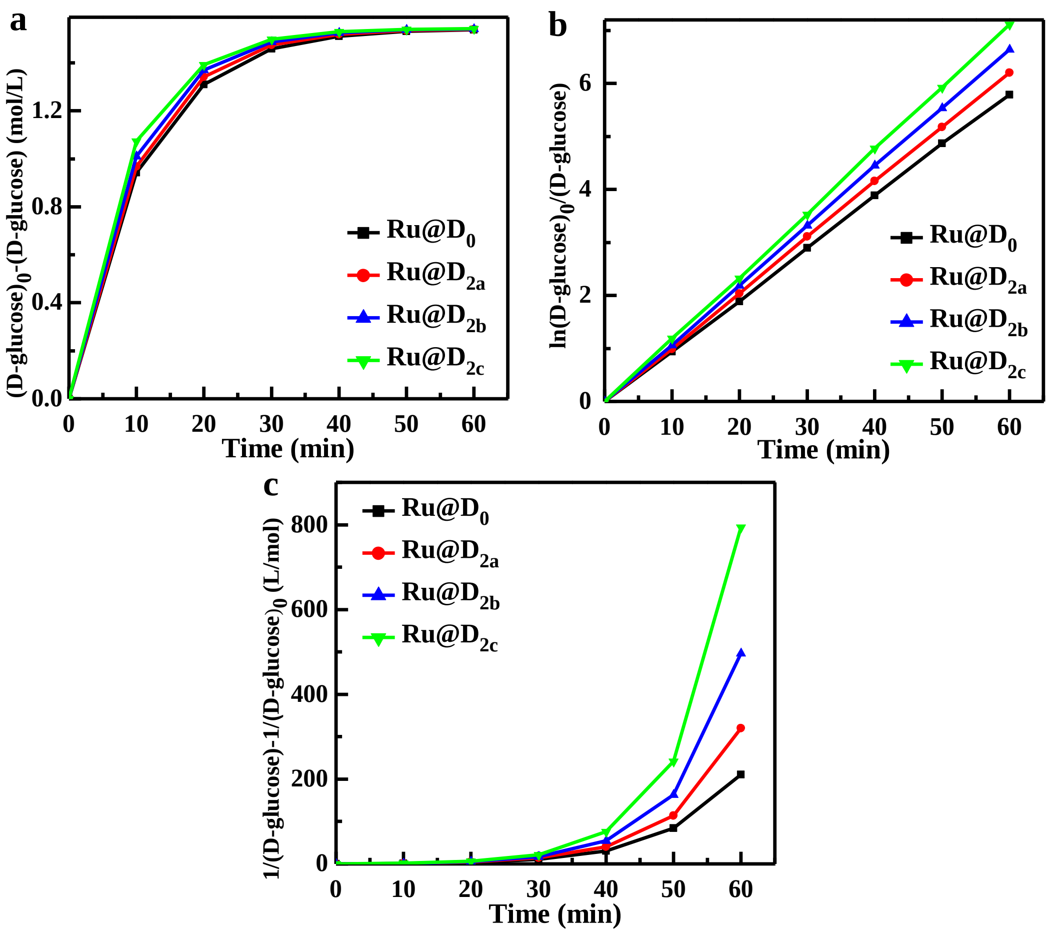

Supplementary Fig. 38 Test D-glucose selective hydrogenation to sorbitol in presence of Ru@D_0 and Ru@$D_{2a\text{-}c}$, respectively, as a zero-order reaction (**a**), the first-order reaction (**b**) and the second-order reaction (**c**). Reaction conditions: D-glucose aqueous solution (25 wt%, 1.543 mol/L, 50 g), catalyst (1 g), H_2 (5.0 MPa), temperature (120ºC), and stirring rate (800 rpm).

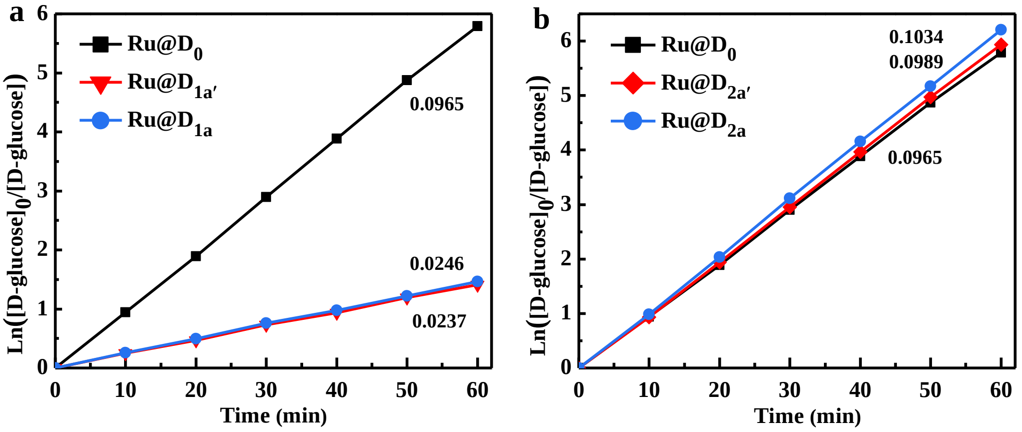

Supplementary Fig. 39 Kinetic studies of D-glucose selectively hydrogenation reactions catalyzed by Ru@D_0, Ru@$D_{1a'}$ and Ru@D_{1a} (**a**), and Ru@D_0, Ru@$D_{2a'}$ and Ru@D_{2a} (**b**). Reaction conditions: D-glucose aqueous solution (25 wt%, 1.543 mol/L, 50 g), catalyst (1 g), H_2 (5.0 MPa), temperature (120 ºC), and stirring rate (800 rpm).

The traditional trial-and-error method based on equations of zero, the first- and second-order reactions are applied to determine the reaction orders of D-glucose selective hydrogenation to sorbitol catalyzed by Ru@D_0, Ru@$D_{1a\text{-}c}$, Ru@$D_{2a\text{-}c}$ catalysts[19]. As shown in Supplementary Fig. 37b, Supplementary Fig. 38b, and Supplementary Fig. 39a-b, the plots of ln[D-glucose]$_0$/[D-glucose] versus time tested as the first-order reactions exhibit linear, illuminating that the D-glucose hydrogenation reactions in presence of Ru@D_0, Ru@$D_{1a'\text{-}c}$, Ru@$D_{2a'\text{-}c}$ follow the pseudo-first order, being consistent with that using Raney nickel[20] and Ru/C as catalysts[21]. Herein, [D-glucose]$_0$ and [D-glucose] are the concentrations of D-glucose at the initial time and at a certain reaction time, respectively. The rate equations of the reactions catalyzed by Ru@D_0, Ru@$D_{1a'\text{-}c}$ and Ru@$D_{2a'\text{-}c}$ (Supplementary Equations 1-9) under the optimized conditions can be obtained based on the defined rate constants *k*:

Ru@D_0 reaction equation:

$$r_{\text{D-glucose}} = -\frac{\mathrm{d}[D\text{-}glucose]}{\mathrm{d}t} = 0.0965\ [D\text{-}glucose]$$ Supplementary Equation 1

Ru@$D_{1a'}$ reaction equation:

$$r_{\text{D-glucose}} = -\frac{\mathrm{d}[D\text{-}glucose]}{\mathrm{d}t} = 0.02365\ [D\text{-}glucose]$$ Supplementary Equation 2

Ru@D_{1a} reaction equation:

$$r_{\text{D-glucose}} = -\frac{\mathrm{d}[D\text{-}glucose]}{\mathrm{d}t} = 0.02456\ [D\text{-}glucose]$$ Supplementary Equation 3

Ru@D_{1b} reaction equation:

$$r_{\text{D-glucose}} = -\frac{\mathrm{d}[D\text{-}glucose]}{\mathrm{d}t} = 0.02736\ [D\text{-}glucose]$$ Supplementary Equation 4

Ru@D_{1c} reaction equation:

$$r_{\text{D-glucose}} = -\frac{\mathrm{d}[D\text{-}glucose]}{\mathrm{d}t} = 0.03882\ [D\text{-}glucose]$$ Supplementary Equation 5

Ru@$D_{2a'}$ reaction equation:

$$r_{\text{D-glucose}} = -\frac{\mathrm{d}[D\text{-}glucose]}{\mathrm{d}t} = 0.\ 09894\ [D\text{-}glucose]$$ Supplementary Equation 6

Ru@D_{2a} reaction equation:

$$r_{\text{D-glucose}} = -\frac{\mathrm{d}[D\text{-}glucose]}{\mathrm{d}t} = 0.1034\ [D\text{-}glucose]$$ Supplementary Equation 7

$Ru@D_{2b}$ reaction equation:

$$r_{\text{D-glucose}} = -\frac{\mathrm{d}[D\text{-}glucose]}{\mathrm{d}t} = 0.1107\ [D\text{-}glucose]$$ Supplementary Equation 8

$Ru@D_{2c}$ reaction equation:

$$r_{\text{D-glucose}} = -\frac{\mathrm{d}[D\text{-}glucose]}{\mathrm{d}t} = 0.1185\ [D\text{-}glucose]$$ Supplementary Equation 9

The related discussions in detail have been given in the section "Influence of two types of defects on catalytic activity" of the main text.

2.3.2. Calculation of Turnover Frequency (TOF)

To evaluate the activity of all these Ru impregnated pristine and defect engineered MIL-100-Cr catalysts, the turnover frequency (TOF) towards the D-glucose selective hydrogenation reaction in presence of these catalysts have been calculated based on Supplementary Equation 10:

$$\text{TOF} = 60\times\frac{r_{\text{glucose}}V_{\text{glucose}}M_{\text{Ru}}}{D_{\text{Ru}}\omega_{\text{Ru}}m_{\text{catalyst}}}$$ Supplementary Equation 10

Where r_{glucose} is the conversion rate of glucose, V_{glucose} is the volume of the aqueous solution containing glucose, M_{Ru} is the molecular mass of Ru, D_{Ru} is the dispersion of Ru NPs in these catalysts obtained from the results of chemisorption, ω_{Ru} is the loading mass percentage of Ru NPs acquired from ICP-OES, m_{catalyst} is the amount of catalyst used in the catalytic reaction. The related discussions about the calculated TOF numbers for these catalysts, listed In Supplementary Table 9, have been given in the section "Influence of two types of defects on catalytic activity" of the main text.

Supplementary Table 9 TOF numbers of Ru@D_0, Ru@$D_{1a'-c}$ and Ru@$D_{2a'-c}$ toward the hydrogenation of D-glucose to sorbitol reaction in relation to that of reported catalysts

Catalyst name	Defective ligand feeding ratios (%)	Rate constant (min^{-1})	Ru adsorbed amount (%)	Metal dispersion (%)	TOF (h^{-1})	Reference
Ru@D_0	0	0.0965	2.711	19.8766	7415.2326	This work
Ru@$D_{1a'}$	5	0.0237	2.493	20.0477	1802.6575	
Ru@D_{1a}	10	0.0246	2.420	19.5389	2150.7299	
Ru@D_{1b}	30	0.0274	2.546	16.1627	2752.4890	
Ru@D_{1c}	50	0.0388	3.044	10.1431	5206.2549	
Ru@$D_{2a'}$	5	0.0989	2.502	21.1571	7495.4229	
Ru@D_{2a}	10	0.1034	2.508	20.8125	8201.4875	
Ru@D_{2b}	30	0.1107	2.589	19.4174	9118.5058	
Ru@D_{2c}	50	0.1185	2.671	14.1507	12978.6005	
5 wt% Ru/C			/		12	[21]
Ni5/SiO_2-R			/		21.6	[22]
Ru/ZSM-5-TF			/		32	[23]
Pt/SBA-15			/		45	[24]
Ni70S			/		50.4	[25]
1%Pt/AC			/		53.1	[26]
Pt/γ-Al_2O_3			/		54	[27]
5Ru/AFPS			/		235	[28]

2.4. The catalytic performances of Ru@D$_0$, Ru@D$_{1a'}$ and Ru@D$_{2a'}$

Supplementary Table 10 The list of D-glucose conversion, selectivity and yield of sorbitol, rate constant (*k*) and turnover frequency (TOF) for Ru@D$_0$, Ru@D$_{1a'}$, Ru@D$_{1a}$, Ru@D$_{2a'}$ and Ru@D$_{2a}$ toward the hydrogenation of D-glucose to sorbitol reaction for the first recycle.

	D-glucose conversion (%)	Selectivity (%)	Yield (%)	Rate constant (*k*)	Turnover frequency (TOF)
RuD$_0$	99.071	89.826	88.991	0.0965	7415.2326
Ru@D$_{1a'}$	98.845	91.529	90.472	0.0237	1802.6575
Ru@D$_{1a}$	99.372	96.331	95.726	0.0246	2150.7299
Ru@D$_{2a'}$	99.283	92.318	91.657	0.0989	7495.4229
Ru@D$_{2a}$	99.343	93.417	92.803	0.1034	8201.4875

Reaction conditions: glucose aqueous solution (25 wt%, 1.543 mol/L, 50 g), catalyst amount (1 g), H_2 (5.0 MPa), temperature (120 ºC), stirring rate (600 rpm) and reaction time (150 min).

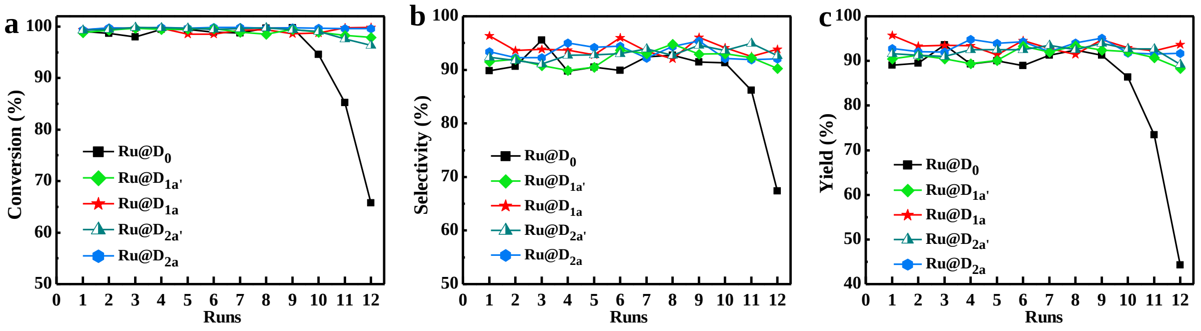

Supplementary Fig. 40 The comparison of D-glucose conversion (**a**), selectivity to sorbitol (**b**) and sorbitol yields (**c**) for the reactions catalyzed by Ru@D$_0$, Ru@D$_{1a'}$, Ru@D$_{1a}$, Ru@D$_{2a'}$ and Ru@D$_{2a}$ for 12 recycles. Reaction conditions: glucose aqueous solution (25 wt%, 1.543 mol/L, 50 g), catalyst amount (1 g), H_2 (5.0 MPa), temperature (120 ºC), stirring rate (600 rpm) and reaction time (150 min) for all Ru impregnated MIL-100-Cr catalysts.

To further find out the optimal feeding ratio of DL*x* to TL (*z*) ranging from 1% to 10%, we have synthesized two types of DEMOFs and Ru@DEMOFs with *z* to be 5%. As shown in Supplementary Fig. 40 and Supplementary Table 10, the D-glucose conversion of Ru@D$_{1a'}$ and Ru@D$_{2a'}$ is comparable to that of Ru@D$_0$, Ru@D$_{1a}$ and Ru@D$_{2a}$. The selectivity to sorbitol and the sorbitol yields of Ru@D$_{1a'}$ and Ru@D$_{2a'}$ are higher than that of Ru@D$_0$, but that of Ru@D$_{1a'}$ and Ru@D$_{2a'}$ are lower than that of Ru@D$_{1a}$ and Ru@D$_{2a}$, respectively. The *k* and TOF of Ru@D$_{1a'}$ and Ru@D$_{2a'}$ are consistent with the expected values deduced from the evolution trends of *k* and TOF numbers upon the increase of incorporated defective ligands. The comparison of D-glucose conversion (a), selectivity to sorbitol (b)

and sorbitol yields (c) for D-glucose selective hydrogenation reaction catalyzed by these catalysts for 12 recycles shows that the recyclability of Ru@$D_{1a'}$ and Ru@$D_{2a'}$ is higher than that of Ru@D_0 but lower than that of Ru@D_{1a} and Ru@D_{2a}, respectively. These results demonstrate that the optimal feeding molar ratio of DLs to total ligands for these two types of Ru impregnated DEMOFs is 10%. The evolution trends of D-glucose conversion, selectivity to sorbitol, sorbitol yields, rate constant and recyclability of Ru NPs impregnated DEMOFs containing type-A and type-B defects with feeding ratio (z) ranging from 0% to 10% are consistent with that of feeding ratio (z) ranging from 0% to 50%, respectively (Fig. 4e-g).

3. Supplementary References

1. Scholten, J. J. F., Pijpers, A. P. & Hustings, A. M. L. Surface characterization of supported and nonsupported hydrogenation catalysts. *Catal. Rev.* **27**, 151-206 (1985).
2. Noei, H. et al. Coverage-induced hydrogen transfer on ZnO surfaces: from ideal to real systems. *Angew. Chem. Int. Ed.* **125**, 2031-2035 (2013).
3. Wang, Y. et al. A new dual-purpose ultrahigh vacuum infrared spectroscopy apparatus optimized for grazing-incidence reflection as well as for transmission geometries. *Rev. Sci. Instrum.* **80**, 113108 (2009).
4. Xu, M. et al. The surface science approach for understanding reactions on oxide powders: the importance of IR spectroscopy. *Angew. Chem. Int. Ed.* **51**, 4731-4734 (2012).
5. Ferey, G. et al. A hybrid solid with giant pores prepared by a combination of targeted chemistry, simulation, and powder diffraction. *Angew. Chem. Int. Ed.* **43**, 6296-6301 (2004).
6. Warren, B. E. X-ray Diffraction Dover publication. (1969).
7. Scherrer, P. Göttinger Nachrichten Math. (1918).
8. Vimont, A. et al. Investigation of acid sites in a zeotypic giant pores chromium(III) carboxylate. *J. Am. Chem. Soc.* **128**, 3218-3227 (2006).
9. Han, M. et al. Immobilization of thiol-functionalized ionic liquids onto the surface of MIL-101(Cr) frameworks by S-Cr coordination bond for biodiesel production. *Colloids Surf., A* **553**, 593-600 (2018).
10. Lieb, A. et al. MIL-100(V) – A mesoporous vanadium metal organic framework with accessible metal sites. *Microporous Mesoporous Mater.* **157**, 18-23 (2012).
11. Marx, S., Kleist, W. & Baiker, A. Synthesis, structural properties, and catalytic behavior of Cu-BTC and mixed-linker Cu-BTC-PyDC in the oxidation of benzene derivatives. *J. Catal.* **281**, 76-87 (2011).
12. Park, J. et al. Introduction of functionalized mesopores to metal-organic frameworks via metal-ligand-fragment coassembly. *J. Am. Chem. Soc.* **134**, 20110-20116 (2012).
13. Diring, S. et al. Controlled multiscale synthesis of porous coordination polymer in nano/micro regimes. *Chem. Mater.* **22**, 4531-4538 (2010).
14. Qiu, L. G. et al. Hierarchically micro- and mesoporous metal-organic frameworks with tunable porosity. *Angew. Chem. Int. Ed.* **47**, 9487-9491 (2008).
15. Sun, L. B. et al. Cooperative template-directed assembly of mesoporous metal-organic frameworks. *J. Am. Chem. Soc.* **134**, 126-129 (2012).
16. Albolkany, M. K. et al. Molecular Surgery at Microporous MOF for Mesopore Generation and Renovation. *Angew. Chem. Int. Ed.* **133**, 14722-14729 (2021).
17. Kim, Y. et al. Hydrolytic transformation of microporous metal–organic frameworks to hierarchical micro‐ and mesoporous MOFs. *Angew. Chem. Int. Ed.* **127**, 13471-13476 (2015).
18. Klimakow, M. et al. Characterization of mechanochemically synthesized MOFs. *Microporous Mesoporous Mater.* **154**, 113-118 (2012).
19. Ahmed, M. J. Kinetics studies of D-glucose hydrogenation over activated charcoal supported platinum catalyst. *Heat Mass Transfer* **48**, 343-347 (2012).
20. Wisnlak, J. & Simon, R. Hydrogenation of glucose, fructose, and their mixtures. *Ind. Eng. Chem. Res.* **18**, 50-57 (1979).
21. Crezee, E. et al. Three-phase hydrogenation of D-glucose over a carbon supported ruthenium catalyst—mass transfer and kinetics. *Appl. Catal., A.* **251**, 1-17 (2003).
22. Schimpf, S., Louis, C. & Claus, P. Ni/SiO_2 catalysts prepared with ethylenediamine nickel precursors: Influence of the pretreatment on the catalytic properties in glucose hydrogenation. *Appl. Catal., A.* **318**, 45-53 (2007).
23. Guo, X. et al. Selective hydrogenation of D-glucose to D-sorbitol over Ru/ZSM-5 catalysts. *Chin. J. Catal.* **35**, 733-

740 (2014).

24. Zhang, X. et al. Platinum-catalyzed aqueous-phase hydrogenation of D-Glucose to D-sorbitol. *ACS Catal.* **6**, 7409-7417 (2016).
25. Kusserow, B., Schimpf, S. & Claus, P. Hydrogenation of glucose to sorbitol over nickel and ruthenium catalysts. *Adv. Synth. Catal.* **345**, 289-299 (2003).
26. Lazaridis, P. A. et al. d-Glucose hydrogenation/hydrogenolysis reactions on noble metal (Ru, Pt)/activated carbon supported catalysts. *Catal. Today.* **257**, 281-290 (2015).
27. Tathod, A. et al. Solid base supported metal catalysts for the oxidation and hydrogenation of sugars. *J. Mol. Catal. A: Chem.* **388-389**, 90-99 (2014).
28. Dabbawala, A. A., Mishra, D. K. & Hwang, J.-S. Selective hydrogenation of D-glucose using amine functionalized nanoporous polymer supported Ru nanoparticles based catalyst. *Catal. Today.* **265**, 163-173 (2016).
